# CRISP: Enhancing ASE Workflows With Advanced Molecular Simulation Post‐Processing

**DOI:** 10.1002/jcc.70384

**Published:** 2026-05-06

**Authors:** Indranil Saha, Daniel Willimetz, Lukáš Grajciar

**Affiliations:** ^1^ Department of Physical and Macromolecular Chemistry, Faculty of Science Charles University Prague Czech Republic

**Keywords:** data analysis, materials science, molecular simulation, python

## Abstract

Molecular simulations are invaluable for analysing molecular systems, but existing post‐processing tools are often limited by a lack of customization, interactivity, and efficiency with large datasets. To address this, we developed CRISP (Comprehensive Repository for Insightful Simulation Post‐Processing), an open‐source Python toolkit designed to enhance workflows within the Atomic Simulation Environment (ASE). CRISP provides a versatile platform for detailed analysis and visualization, featuring a customizable and modular design, various static analysis methods, interactive 3D visualizations, and parallel processing capabilities optimized for high‐performance computing. We demonstrate its effectiveness through case studies, including the analysis of statistical convergence in zeolite simulations, subsampling large datasets for machine learning, and analysing the dynamic stability of atomic clusters. CRISP effectively bridges the gap between raw simulation data and actionable insights, offering an efficient solution for researchers and saving significant time in code development.

## Graphical abstract

CRISP is an open‐source, modular Python post‐processing toolkit that bridges raw molecular simulation data and actionable scientific insights by extending ASE with parallelised analysis workflows, including DBSCAN clustering, SOAP‐FPS subsampling, statistical error analysis, and interactive 3D visualisation, demonstrated on zeolite, platinum cluster, and bulk water systems.

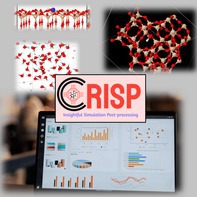

## 1 Introduction

Molecular simulations have become invaluable tools for modelling and analysing the behavior of molecular systems, often serving as a means to prove or disprove various hypotheses, leading to significant discoveries in the fields of biology, chemistry, and materials science [1, 2, 3, 4]. Recent advances in computational power, driven by state‐of‐the‐art algorithms and increased accessibility of high‐performance computing (HPC) resources, have revolutionized the field of molecular simulations [5]. Today, atomistic simulations routinely involve systems comprising thousands of atoms modelled over extended timescales [6]. The development of reliable software packages and efficient computational methods, such as those based on machine learning, has allowed the capture of unexpected dynamical phenomena [7, 8, 9, 10]. For example, in the field of materials science [11], we have seen important discoveries regarding the defect dynamics [12, 13, 14], theoretical spectroscopy [15, 16, 17, 18] and interfacial interactions with unprecedented accuracy [19, 20, 21]. However, these works have provided us with both voluminous and complex data [22], and to fully understand the detailed data, trends, and correlations embedded in those simulations [23, 24], more nuanced and integrated post‐simulation analysis and visualization tools are needed [25, 26, 27, 28].

The Atomic Simulation Environment (ASE) is a Python‐based framework designed for the setup, manipulation, and analysis of atomistic simulations [29]. It has a widespread usage within the community of physical chemists and multidisciplinary materials scientists. ASE provides a robust infrastructure for simulation management, but post‐processing analysis of the high‐volume trajectory data (e.g., a system with ≳1000 atoms producing $≳10^{5}$ structural frames over only a few ns of molecular dynamics simulations, which occupies a few GB) generated by modern high‐performance computing (HPC) resources presents specific technical challenges. Although dedicated platforms such as OVITO [30] and MDAnalysis [31] are available, it remains difficult to natively integrate the highly interactive or parallelized workflows often necessary for the effective analysis of detailed trajectories directly within the ASE ecosystem. Alternatively, standard Radial Distribution Functions (RDF) or Mean‐Squared Displacement (MSD) implementations as implemented in ASE often lack modularity to isolate subsystem selections or to exploit multi‐core parallelism at scale. Parallelized SOAP–Farthest Point Sampling (SOAP‐FPS) for ML‐ready subsampling and built‐in error analysis are generally missing across these tools. This motivates CRISP as an integrated toolkit specifically designed for materials scientists, machine learning practitioners, and high‐performance computing users within the computational chemistry community. It extends ASE with advanced clustering, interactive 3D volumetric analysis, SOAP‐FPS subsampling, and parallelized, uncertainty‐aware processing routines. We discuss the scientific justification and comparative mapping of CRISP versus other analysis packages in a dedicated Supporting Information: Section 1 with a short comparison provided in a Table S1.

Inspired by the tight coupling of simulation and analysis in GROMACS [32], a package heavily used in studying biological systems, our goal was to build an analysis toolkit that bridges this gap in materials science by offering various useful analysis modules to expand the infrastructure of the Atomic Simulation Environment (ASE) [29]. Thus, the aim of this work is to enable users to examine complex systems while benefiting from the ASE's existing ecosystem for executing simulations and a well‐documented Application Programming Interface (API).

CRISP (Comprehensive Repository for Insightful Simulation Post‐Processing) provides an open‐source toolkit for advanced molecular simulation data analysis and visualization. The features offered in CRISP include the following:

1. *Customizable input and modules*: Allows optional inputs for specific needs and allows the module's functionality to be extended in the existing workflow.
2. *Various methods of static analysis*: Clustering using advanced algorithms like DBSCAN, visualization and analysis of hydrogen bonds using graph theory [33], 3D volumetric data visualization of atoms, sub‐sampling and statistical analysis of simulation data, and other methods are available.
3. *Interactive analysis*: In the interactive figures (generated as HTML files) of the visualized data, users can isolate and probe the regions or atoms of interest.
4. *Parallel processing*: Optimized for HPC, enabling efficient analysis of large trajectories.
5. *Unified ASE workflow and data handling*: Ensures compatibility with ASE, allowing a seamless workflow and data conversion to other formats.

**FIGURE 1:**
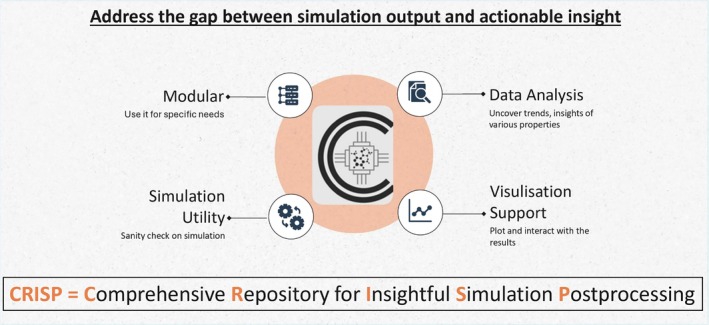
CRISP's role in bridging raw simulation data to actionable insights. The modular architecture enables targeted workflows for simulation diagnostics (utility), automated analysis of properties (data), and interactive visualization.

By integrating these features into a single package (see its schematic depiction in Figure 1), CRISP aims to provide a versatile, efficient and interactive platform for detailed post‐simulation analysis of simulation data. Ideally, this platform should allow researchers to save valuable time in code development and aid in analysing their systems.

## 2 Software Description

CRISP is implemented as a modular Python package [34] designed to ensure flexibility (see Figure S1) and ease of extension, alongside being easily maintained. Since Python, as a programming language, benefits from its extensive ecosystem of scientific libraries, readability, and widespread usage in research communities, it also makes the source code compatible with ASE [29].

**FIGURE 2:**
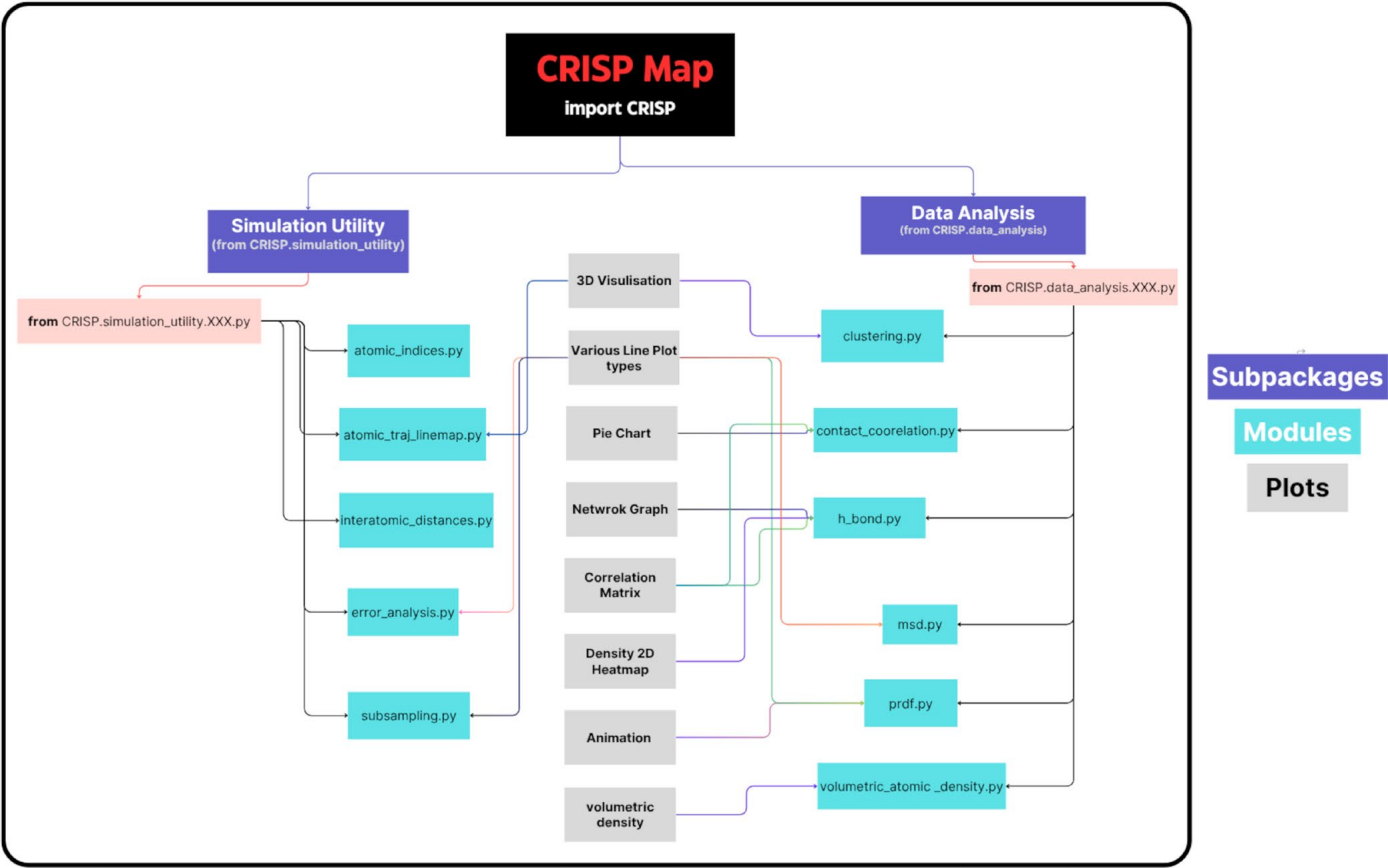
The diagram provides a concept map of CRISP, distinguishing two main branches—Simulation Utility and Data Analysis, which together feed into various visualization capabilities, ranging from simple line plots to advanced 3D renderings.

Figure 2 is a concept map that provides a structural overview of CRISP, highlighting its two sub‐packages and plotting tools offered. From both the sub‐packages, various modules offering different tasks can be used, and in most cases, they produce a plot as part of the analysis. The core of scientific analysis and computational workflows is implemented using the Python programming language [35]. In total (see Figure S2 for detailed source code characteristics via the package **cloc** [36]), we have 47 original Python files, with approximately 7370 lines of code and 2669 comments. The comment to code ratio is 36%, reflecting that the code is thoroughly explained in doc‐strings for the users to understand the parameters used, and the logic of the function, thereby allowing the end‐user to handle errors or expand the code easily for specific needs further.

CRISP is available on PyPI and can be easily installed via pip. The package has been tested on Python 3.9–3.11 across Linux, macOS, and Windows platforms, and is continuously validated by automated GitHub Actions (including Dependabot‐driven dependency checks) to ensure installations remain reliable. Further details of the installation can be found in the installation section of the website, https://crisp.readthedocs.io/en/latest/. A typical installation and verification workflow is shown below: pip install crisp-asepip install pytest pytest-cov coveragepython -m pytest --pyargs CRISP -v


Alternatively, users can install directly from the GitHub repository:git clone https://github.com/Indranil17/CRISP.git cd CRISP pip install .pip install pytest pytest-cov coveragepython -m pytest --pyargs CRISP -v


Both installation methods automatically resolve all runtime dependencies (ASE, scikit‐learn, seaborn, plotly, pandas, networkx, statsmodels, fpsample). Installing pytest enables running the full test suite.

After installation, users can confirm the successful setup by running pythonic **test** [37] as pytest–pyargs CRISP‐v. This executes a comprehensive suite of tests designed to validate standard functionality, edge cases and numerical stability across the entire codebase. Consequently, the package achieves 93% code coverage (see Figure S3), which ensures that all primary modules within the simulation utility and data analysis subpackages are robustly verified for research applications.

Detailed installation instructions, user guides, and tutorials are available on our website:

https://crisp.readthedocs.io/en/latest/. Also in Supporting Information: Section 2.1, we provide the details on the introductory and advanced tutorials. The code is fully available in Zenodo at DOI: 10.5281/zenodo.16990590 and serves as a permanent backup of the original GitHub repository at, https://github.com/Indranil17/CRISP, where users can report issues, contribute to development or provide necessary feedback.

### 2.1 Architecture

The two sub‐packages of CRISP are designed for two different purposes, Simulation Utility is mostly used to preprocess the simulation data that can be then used for further analysis or validation of molecular dynamics, for example, for monitoring the atomic movements (positions, velocities) during simulations. Data analysis is purely dedicated to extracting quantitative insights from simulation outputs. Its goal is to find intricate patterns, correlations and other possible insights from the input. Here, we analyse much of the chemistry and interactions of the atoms.

The Structural Overview diagrams provided in Figures S4 and S5 illustrate the internal organization and various functions within the individual modules. This representation emphasizes the modular flexibility of CRISP, as users can chain distinct operations—such as subsampling, clustering and radial distribution function (RDF) analysis—or isolate specific functions for targeted applications while maintaining compatibility with ASE and high‐performance computing (HPC) environments. In Supporting Information: Section 1.3, we provide an example case to highlight the ability of CRISP (its functions) to be modulated for on‐the‐fly analysis (see Figure S1). In the upcoming sections, we dive into understanding the implementation of different modules, starting with Section 2.2, where we explain the functionalities of the modules for each sub‐package, and also we explore the Application Programming Interface (API) design and input/output conventions details with the **help** function.

One of the important dependencies of the package is the *joblib* library [38], which provides essential parallel computing capabilities for processing large‐scale simulation trajectories. These tools enable efficient distribution of computationally intensive tasks across multiple CPU cores, dramatically accelerating trajectory analysis workflows.

**FIGURE 3:**
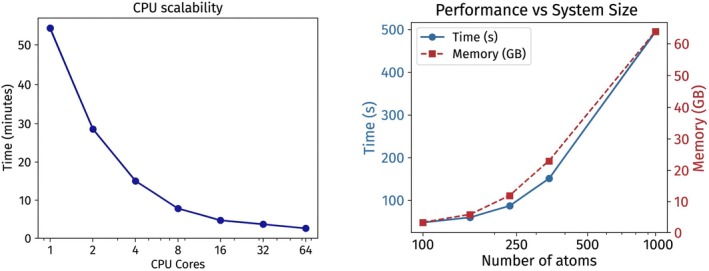
(Left) CPU scalability shows a substantial reduction in computation time with the increasing number of CPU cores. (Right) Time and memory usage as a function of system size at 32 CPUs. Benchmark systems are zeolites with water molecules, and larger sizes are generated using their supercells.

We test the efficiency of parallel implementation for the “contact_correlation.py” module calculating average oxygen coordination number in water‐loaded zeolite systems. As shown in Figure 3, the computation time decreases significantly with the number of CPU cores due to the parallel execution of analysis tasks in CRISP, demonstrating near‐ideal parallel scaling (log–log slope ≈−0.81). Additionally, the performance benchmark on various hydrated zeolite systems shows that both execution time and memory requirements increase with system size, as expected. The scaling is close to linear (log–log slope ≈1.1) due to efficient parallelization, and the use of cutoffs that reduce the number of interatomic distance calculations. This highlights CRISP's capability to handle large‐scale datasets when adequate computational resources are available efficiently.

A similar performance acceleration is demonstrated for radial distribution function analyses (the prdf.py module), where a direct comparison between the native ASE and CRISP reveals that CRISP achieves a 1300‐fold cumulative speedup on 64 cores (and a 40‐fold algorithmic speedup on a single core). These are detailed in Supporting Information (see Table S2; Figure S21).

### 2.2 Functionalities and API Design

Since the goal was to make the API of CRISP intuitive, we named each module in a self‐explanatory manner after the type of analysis it performs on simulation data. As illustrated in the structural overview in Figure 2, CRISP is divided into two primary branches: Simulation Utility and Data Analysis. The Simulation Utility subpackage provides tools for trajectory preprocessing and statistical validation, including **atomic_indices.py** for coordinate extraction, **interatomic_distances.py** for distance matrix management and **subsampling.py** for identifying diverse structures using SOAP descriptors. This branch also features **error_analysis.py**, which quantifies uncertainty via autocorrelation and block averaging, as well as **atomic_traj_linemap.py** for interactive 3D movement paths. Complementing these utilities, the Data Analysis subpackage extracts quantitative chemical insights. Key modules include **clustering.py** for identifying structures using the DBSCAN algorithm, **contact_correlation.py** for coordination number analysis based on van der Waals radii and **h_bond.py** for investigating hydrogen bond networks using geometric criteria. For dynamic properties, **msd.py** calculates diffusion coefficients via Einstein's relation, while **prdf.py** and **volumetric_atomic_density.py** provide spatial correlation analysis and volumetric 3D density mapping, respectively.

A brief overview of all the functions and their detailed mathematical derivations for these functions is provided in Supporting Information: Section 2.3 and 2.4, respectively.

**FIGURE 4:**
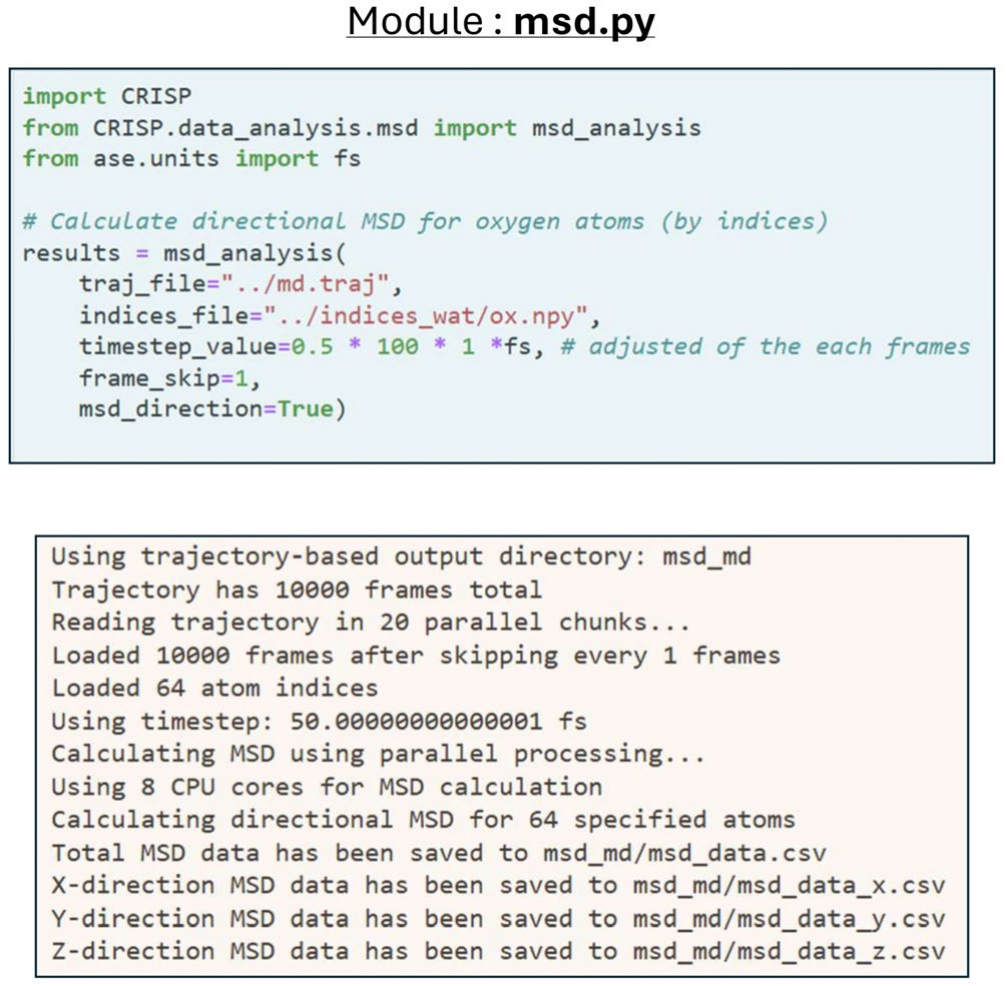
Standardized Input–output workflow for MSD analysis in CRISP. (**Top**) Code invocation with standardized parameters: traj_file (Path to the ASE trajectory file), frame_skip (Number of frames to skip between samples), and other available parameters are chosen. (**Bottom**) Automated parallel execution and structured output generation, including CSV files (folder locations).

To ensure a seamless user experience, CRISP adopts a unified naming convention across all modules. This design ensures that workflows are easy to memorize and execute, as the keyword names stay consistent across different modules. Furthermore, the package employs a standardized input and output workflow (Figure 4), while standardizing units and automatically managing parallel processing via the joblib library (see Supporting Information: Table S2 and Figure S21). Most modules additionally offer automated plotting options to interactively visualize results, which are saved in accessible formats like CSV and HTML.

**FIGURE 5:**
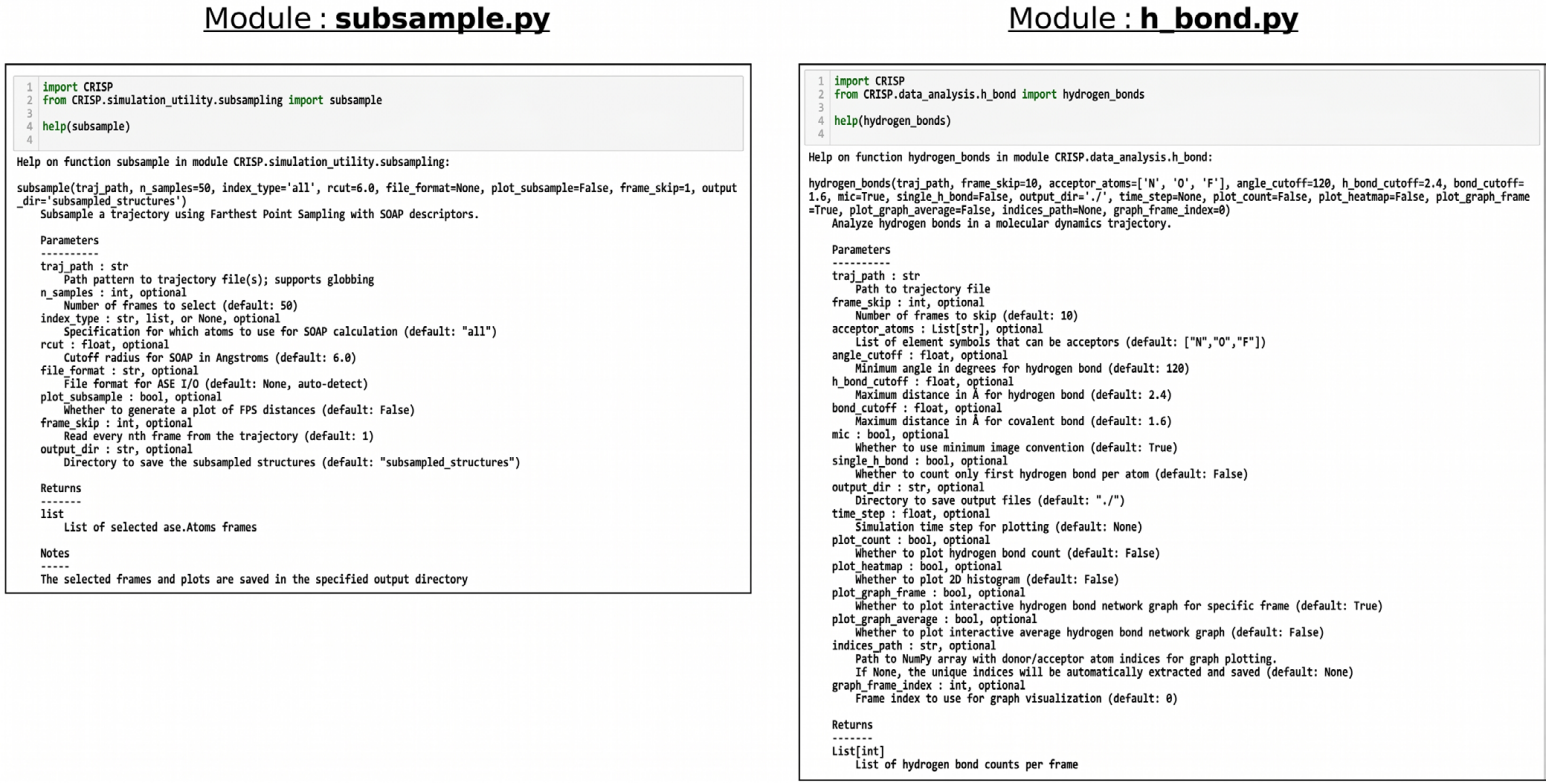
API consistency and modular design of CRISP. The comparison between the subsampling module (*left*) and hydrogen bond analysis module (*right*) demonstrates the use of identical parameter naming conventions such as traj_path, frame_skip and output_dir. This uniformity enables users to apply similar keywords across different algorithmic tasks, significantly reducing the learning curve for new analysis workflows.

These examples highlight that the code has unified parameter naming, standardized units and inputs (e.g., time in femtoseconds, distances in angstroms), and structured outputs. Also, as part of most of the outputs from various functions, there is an option (see, for example, the parameters listed in Figure 5) to plot or interactively visualize the results, with the plots and results being automatically saved.

### 2.3 Schematic Workflow Example

We schematically outline the standard CRISP workflow for a case study, in which different functions from the modules would be used systematically to provide relevant insights into the system at hand. The case system is the water‐loaded aluminosilicate zeolite with the molecular trajectory generated using the neural network potential [39]. The system considered is zeolite faujasite (FAU) with Si/Al = 3 (12 aluminum) and 48 waters in the simulation cell. We ran the NVT molecular dynamics simulation at a temperature 300 K for a nanosecond (ns) with a 0.5 femtosecond (fs) timestep [14].

**FIGURE 6:**
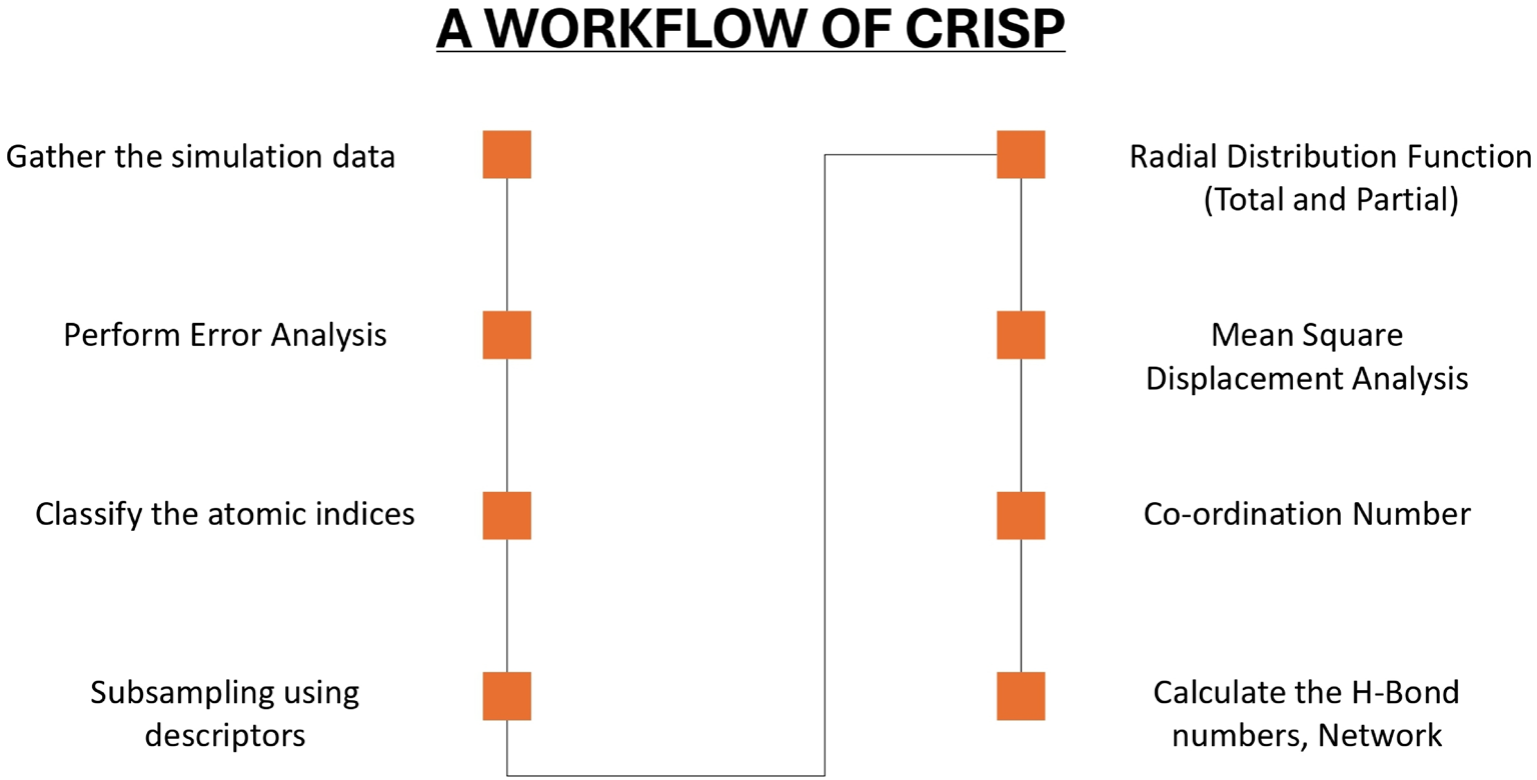
CRISP workflow for water‐loaded faujasite (FAU) zeolite (Si/Al = 3, 48 H_2_O molecules), The following analyses we performed using sequentially: CRISP.simulation_utility modules starting with (**Left**) (1) error analysis on the energy and temperature; (2) indices generation for classification of the atoms; (3) subsampling of the structures and then following with the CRISP.data_analysis branch modules to calculate (**Right**) (4) **prdf** for (O—O, O—H, H—H) pairs; (5) **msd** of water oxygens; (6) **cn** on water oxygen; and (7) **h‐bond** calculation with oxygen as donor atoms.

The tasks performed on this trajectory, as seen in the workflow diagram in Figure 6, would entail first using a set of modules from *simulation utility* branch (Supporting Information: Section 2.3.1) like atomic indices or error analysis and then the modules from *data analysis* branch (Supporting Information: Section 2.3.2) are deployed to obtain mean square displacement (msd), partial radial distribution functions (prdf), coordination numbers (cn), and hydrogen bond counts (h‐bond).

This complete workflow in Figure 6 executes in *124 s* on 4 cores (standard laptop with Intel Core i7‐1065G7 @1.50 GHz) in a single interactive Jupyter notebook, with the Jupyter notebook available at the CRISP Github example/Workflow/collage_calculations.ipynb for further details, testing and reproduction.

## 3 Illustrative Examples

Now as part of the demonstration, we put the capabilities of the individual modules of CRISP through benchmarking and analysis for diverse molecular systems, specifically for error analysis (Aluminosilicate MFI zeolite), subsampling (small organic molecules), clustering (platinum in CHA zeolite), and mean square displacement, coordination number and hydrogen bonding (bulk water).

### 3.1 Case Study: MFI Zeolite, Error Analysis

Assessing statistical convergence and uncertainty in time‐dependent simulations is essential to draw reliable conclusions. During long molecular dynamics or Monte Carlo runs, it is not always evident whether the simulation has fully converged or how reliable the reported averages are. A common approach is block averaging, which provides a rough error estimate by dividing the trajectory into fixed‐size blocks [40]. However, this method is sensitive to the choice of block size. An alternative is to analyse the autocorrelation function of observables, which also offers a way to quantify statistical inefficiency and evaluate convergence [41]. Recently, the autocorrelation function in analysis has gained further interest as a robust approach to quantify statistical inefficiencies and assess convergence [42]. The implementation of time‐dependent data analysis in CRISP automatically determines the optimal block size based on error convergence and enables straightforward estimation of standard errors and convergence through autocorrelation analysis.

#### 3.1.1 Error and Convergence

To illustrate the usefulness of the time‐dependent statistical analysis implemented in CRISP, we revisit previous work focused on the prediction of ^27^Al chemical shifts in zeolite MFI [15]. This study reported 1 ns molecular dynamics trajectories, from which chemical shifts were predicted for subsampled structures.

First, we examine the statistical convergence of the molecular dynamics simulation. In Table 1, we report the standard error of the mean (SEM) of the total energy over different trajectory lengths, computed using both the autocorrelation function (ACF) and block averaging. As expected, the standard error decreases with increasing simulation length, highlighting the benefit of longer trajectories for improved statistical reliability. At 1 ns, the SEM falls below 1 kJ/mol, indicating that the system is converged. In contrast, short trajectories, such as those of 10 ps or less, yield significantly larger uncertainties, particularly when estimated using the autocorrelation function.

**TABLE 1:** Standard error of the mean (σ) calculated using the autocorrelation function (ACF) and block averaging (block) for MFI (Si/Al = 91, 3 water molecules per Al atom).

Time [ps]	σ (ACF) [kJ/mol]	σ (block) [kJ/mol]
1	64.3	8.7
10	31.7	6.9
100	4.6	3.4
1000	0.8	1.2

The autocorrelation‐based estimate is more sensitive than block averaging, especially at shorter timescales. This is because the ACF explicitly accounts for temporal correlations in the data, which can persist over several picoseconds [43]. The ACF integrates over the full correlation time of the observable, offering a physically interpretable estimate of the true statistical uncertainty.

**FIGURE 7:**
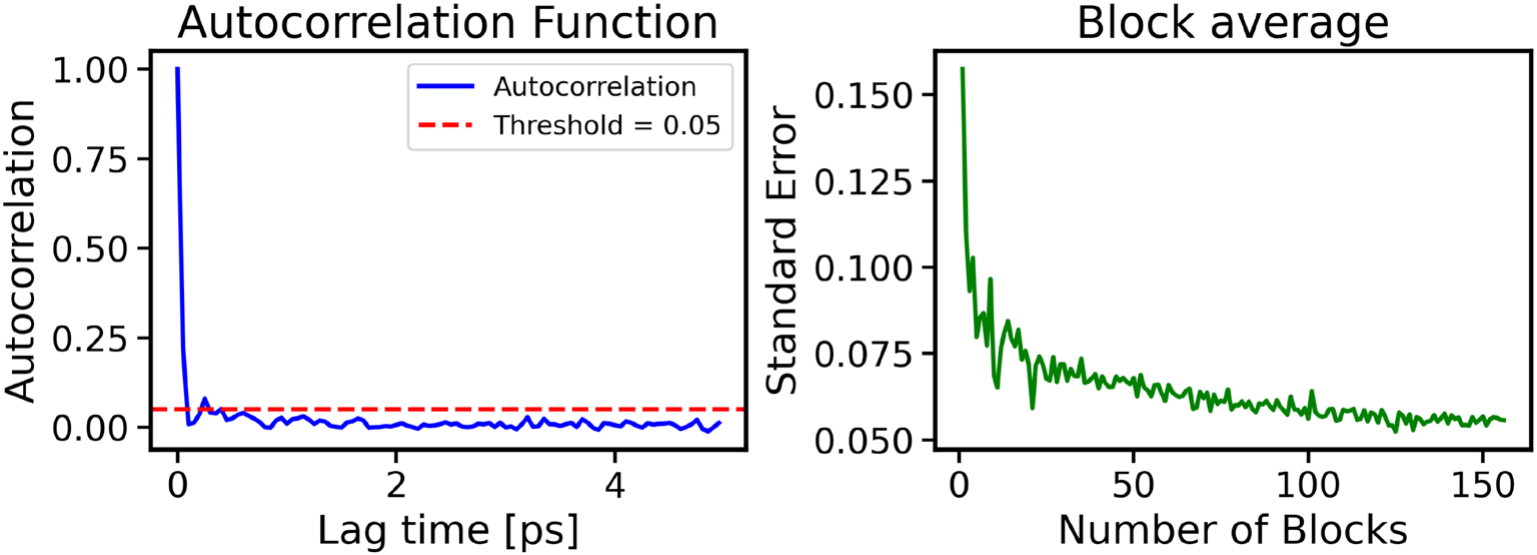
Left: Autocorrelation function for the ^27^Al chemical shift in zeolite MFI from a 1 ns simulation. Lag time corresponds to the MD simulation time between data points used to compute time‐separated correlations. Right: Standard error of the ^27^Al chemical shift in ppm as a function of the number of blocks used to divide the unprocessed data during block averaging.

Second, the same analysis is performed on the ^27^Al chemical shift in the same MD simulation. The autocorrelation time for the ^27^Al chemical shift is determined to be 50 fs (Figure 7), indicating that it equilibrates relatively quickly. The error is less than 0.05 ppm according to both the autocorrelation function and block averaging methods, suggesting that a 1 ns simulation is sufficient to reliably predict the ^27^Al chemical shift in zeolite MFI. The convergence of both the autocorrelation function and the block size is shown in Figure 7, illustrating how the error decreases as the simulation time increases.

### 3.2 Case Study: rMD17 Dataset, Dataset Subsampling

Modern atomistic modelling produces large volumes of structural data, especially from extensive molecular dynamics simulations and structure searches. Although these datasets are rich in information, they often contain substantial redundancy, with many configurations offering little new structural or chemical insight. This has recently become a particularly acute problem for training of machine learning potentials, as the training on these large unfiltered datasets can be both inefficient and may reduce model generalization. To alleviate this, various subsampling strategies can be adopted, and in this case study, we apply a SOAP‐descriptor based subsampling strategy. This approach selects a compact and diverse subset of structures that preserves the essential chemical variability of the full dataset. The reduced set enables more efficient and focused training of neural network potentials without compromizing predictive performance.

#### 3.2.1 SOAP‐FPS Example

To demonstrate the practical benefits of subsampling, we selected the rMD17 dataset as a benchmark. The revised MD17 (rMD17) dataset comprises gas‐phase molecular dynamics trajectories of small organic molecules with energies and forces computed at the PBE level, providing on the order of 100,000 configurations for benchmarking machine‐learned potentials [44]. All molecular trajectories from the constituent systems were first combined into a single aggregated pool of atomic structures. We then computed SOAP descriptors [45] for each structure and applied furthest point sampling (FPS) to sample diverse structures. This process ranks configurations by their dissimilarity in descriptor space, allowing us to systematically select the most representative and distinct structures. From this ranked list, we extracted the 10,000 most diverse configurations as a reduced training set. To assess the impact of this subsampling, we trained the MACE [9] model on both the FPS‐selected data and a standard 10,000 structure subset randomly chosen according to the original rMD17 protocol. For both cases, we used consistent hyperparameters: 128 channels, a radial cutoff of 6 Å, and invariant messages only. The training procedure included an early stopping criterion with a patience of 30 epochs, terminating once validation performance plateaued. The model trained on the standard rMD17 subset required 144 epochs to converge, whereas the FPS‐reduced set reached full convergence in only 66 epochs. This reduction in training time highlights the effectiveness of descriptor‐guided subsampling in accelerating model development without compromizing structural coverage.

**FIGURE 8:**
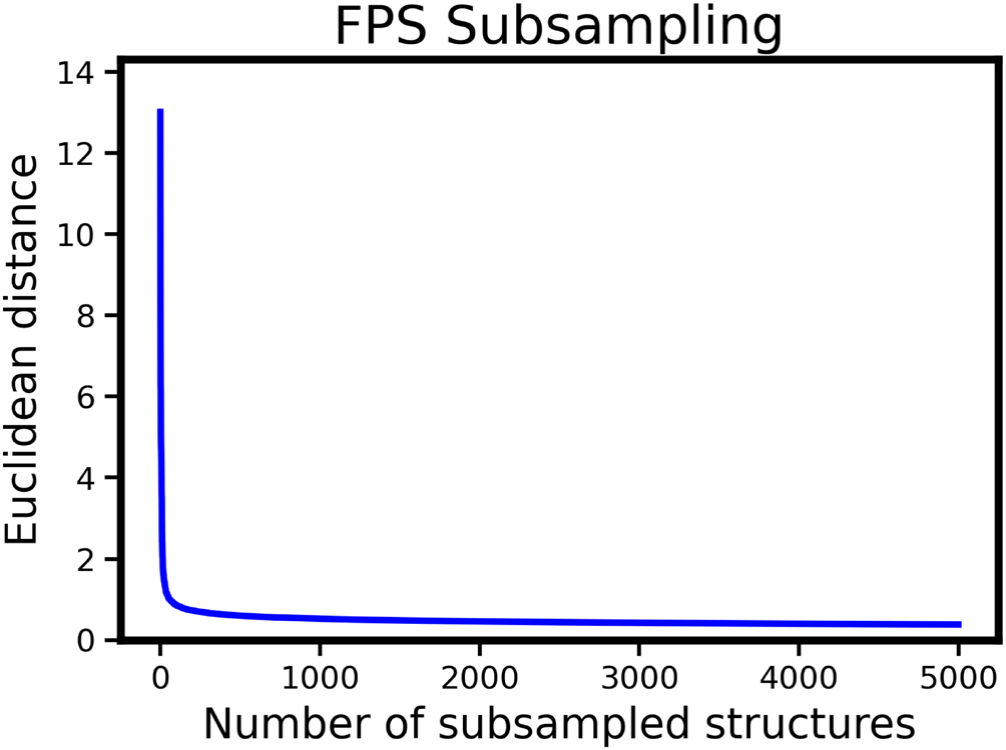
The diminishing diversity in structures sampled from rMD17 database using furthest point sampling based on SOAP descriptors of the rMD17 structures.

Furthermore, due to the redundancy inherent in the rMD17 database, selecting 10,000 structures from the original 50,000 may still result in a redundancy. To assess the convergence of the sampling process, as part of the CRISP code, a figure can be generated to visualize the sampling process (Figure 8). From the figure, it is evident that convergence occurs much earlier. To assess the efficiency of the sampling, the MACE model was trained using only 1000 structures. Remarkably, this model converged within 137 epochs, while maintaining the accuracy of the model trained on the full set of 10,000 structures (see Supporting Information: Section 3.2 and Table S3 for more details).

While this subsampling technique is useful for training machine‐learned potentials, it can also serve as a pre‐screening tool for molecular dynamics or Monte Carlo simulations by selecting the most diverse structures from a given trajectory or dataset.

### 3.3 Case Study: Platinum Migration in Zeolites

This case study focuses on re‐analysing molecular dynamics (MD) simulation data from Heard et al. [19], who investigated the migration of platinum atom (Pt_1_) and clusters (Pt_3_, Pt_5_) within the small‐pore CHA zeolite. Their original work utilized custom reactive neural network potentials (NNPs), trained at the PBE + D3 level of theory, to perform long‐timescale (~25 ns) simulations at elevated temperatures (750, 1000, and 1250 K). These simulations employed a 1 fs time step and a Nosé–Hoover thermostat. Here, we apply two modules from the CRISP toolkit to the trajectory data to get the volumetric atomic density mapping and carry out DBSCAN analysis on platinum clusters.

#### 3.3.1 Clustering

##### 3.3.1.1 DBSCAN Parameter Details

To employ the DBSCAN clustering algorithm [46], we must define two key parameters: the distance cutoff (ϵ) and the minimum number of points/atoms required to form a cluster (“min_samples”). For the minimum cluster size, we set “min_samples” to 2 atoms, ensuring the minimum for a cluster. For the distance threshold, we selected two values based on the Pt–Pt radial distribution function (RDF) analysis of the MD trajectories (Supporting Information: Figure S6). The RDFs show a sharp, well‐defined peak at approx. 2.46 Å across all temperatures and cluster sizes, indicating strong Pt–Pt bonding. This justifies the choice for a first tight distance threshold at ϵ=2.6Å. To also explore the effect of a slightly looser bonding while still focusing on bonded atoms, we also used a more looser threshold at ϵ=3.0Å. We employed DBSCAN clustering with these two distance thresholds (ϵ=2.6 and 3.0Å) and “min_samples = 2” to analyse the dynamics of Pt_3_ and Pt_5_ clusters within the CHA zeolite framework. This dual‐threshold approach allows us to assess cluster integrity and fragmentation under slightly different definitions of connectivity.

**FIGURE 9:**
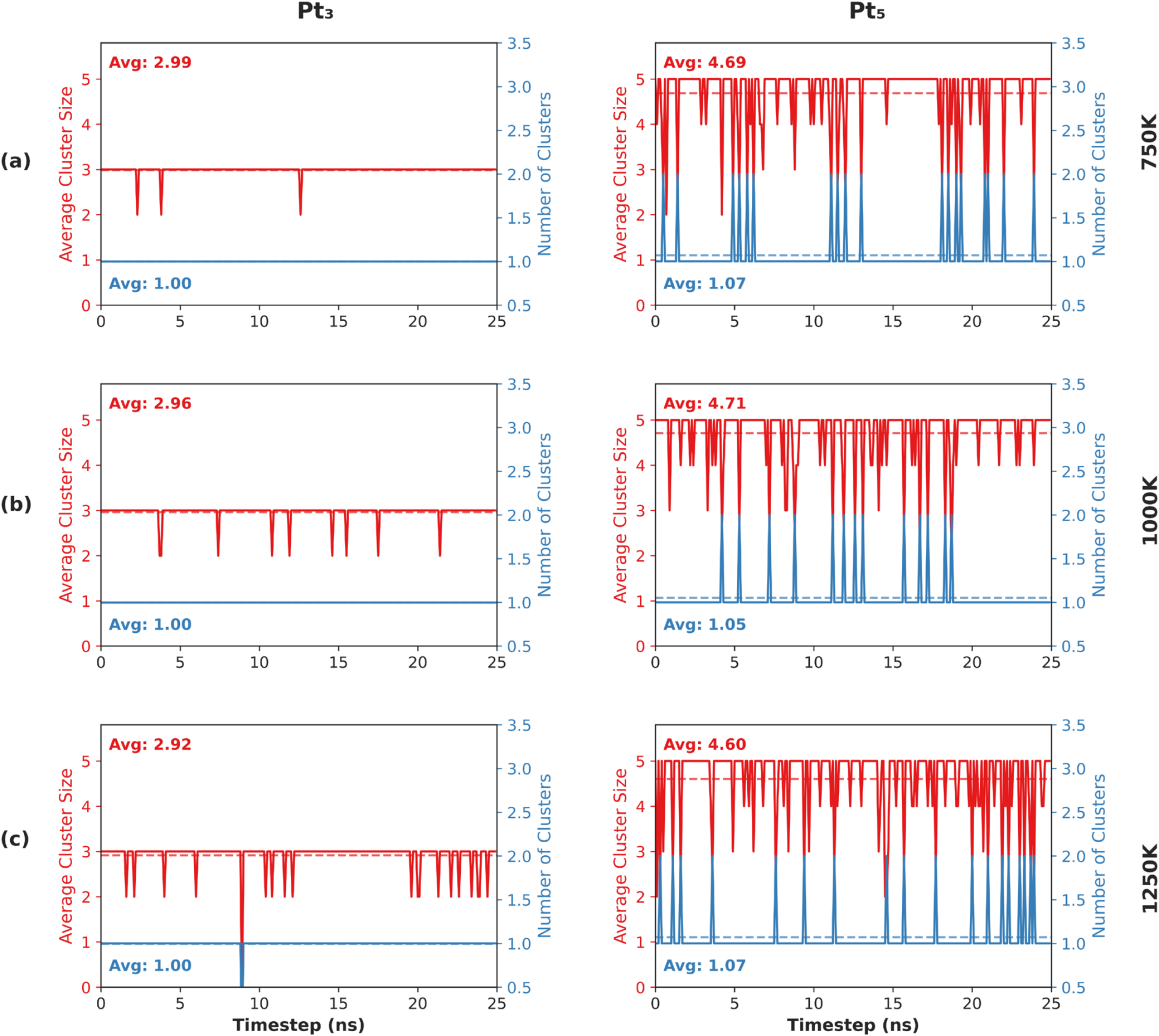
DBSCAN clustering analysis results for Pt_3_ (left column) and Pt_5_ (right column) clusters in CHA zeolite using a distance threshold ϵ=2.6Å and “min_samples = 2.” Each plot shows the average cluster size (red, left *y*‐axis) and the number of clusters (blue, right *y*‐axis) as a function of simulation frame number at (a) 750 K, (b) 1000 K, and (c) 1250 K. Dashed lines indicate the average values over the trajectories, with corresponding numerical values displayed in matching colors. In frames where isolated atoms are present (i.e., not forming clusters given the chosen DBSCAN parameters), these are treated as outliers by the DBSCAN algorithm and are not considered in the average values of cluster sizes plotted here. This results in non‐integer average cluster sizes. However, CRISP is flexible, thus single‐atom outliers (these data are reported in the txt and pkl) can be included in the analysis if and when a more comprehensive representation of the data is needed.

The key findings from the clustering analysis are illustrated in Figure 9 for the primary distance threshold (ϵ=2.6Å). The corresponding results using the looser threshold (ϵ=3.0Å) are presented in Figure S7 for comparison.

- Pt_3_ Clusters:
  – At ϵ=2.6Å: The Pt_3_ cluster demonstrates high stability across all temperatures (Figure 9, left column). It predominantly exists as a single cluster (average number of clusters ≈1.0), with an average size consistently close to 3.0. Only rarely, transient fragmentation events occur, where the average size briefly drops, likely corresponding to momentary dissociation into Pt_2_ + Pt_1_ during interactions with the zeolite framework as seen in Figure S8; then it is followed by rapid recombination. These events become slightly more frequent at higher temperatures but remain infrequent overall.
  – At ϵ=3.0Å: Using the looser cutoff, the Pt_3_ cluster appears even more stable, remaining as a single entity for the whole simulation time (Figure S7, left column).
- Pt_5_ Clusters:
  – At ϵ=2.6Å: The Pt_5_ cluster exhibits significant dynamic instability and frequent fragmentation at all simulated temperatures (Figure 9, right column). The average cluster size frequently drops well below 5.0, and the number of clusters often exceeds 1, indicating dissociation into smaller fragments in Figure S8 (e.g., Pt_4_ + Pt_1_, Pt_3_ + Pt_2_). While it remains predominantly as a single cluster (~1.05–1.07), the fluctuations reveal a highly dynamic system where fragmentation and recombination are rather common.
  – At ϵ=3.0Å: Similar to Pt_3_, the Pt_5_ remains as a single cluster across all temperatures.

Consistent with the original study, the analysis using the larger threshold (ϵ=3.0Å) indicates that both Pt_3_ and Pt_5_ clusters behave largely as single, cohesive entities, suggesting the atoms, despite a sizeable structural fluctuation, stay in close proximity. However, using a tighter threshold (ϵ=2.6Å), one may recover interesting details about the internal cluster Pt‐Pt bond dynamics, for example, that the Pt_3_ cluster remains rather rigid, whereas the Pt_5_ cluster is considerably less stable, readily undergoing internal rearrangements and fragmentation.

#### 3.3.2 Volumetric Atomic Density

**FIGURE 10:**
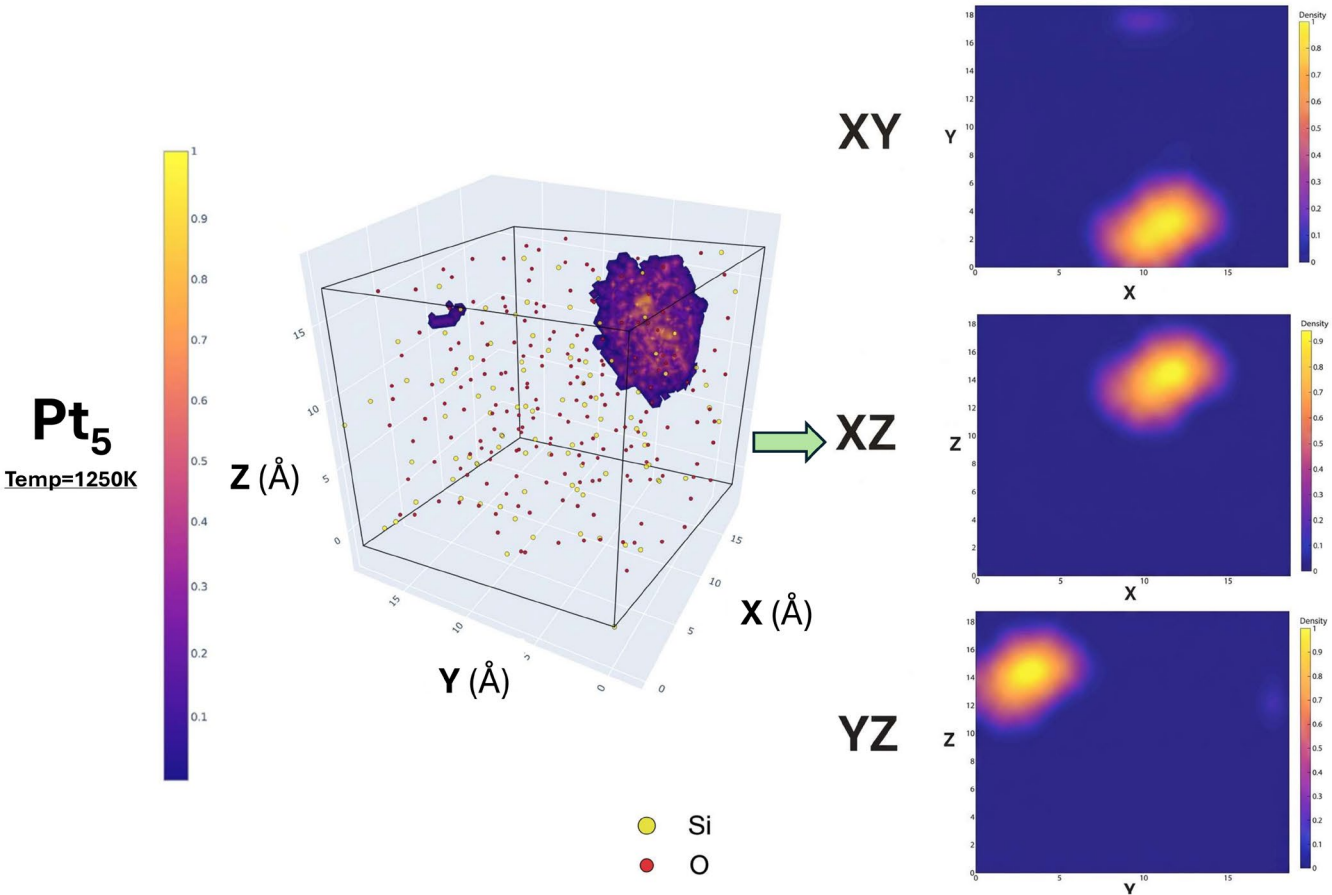
Volumetric density map of Pt_5_ (colored clouds) within the CHA zeolite framework (Si: yellow spheres, O: red spheres) at temperature 1250 K. The atomistic probability density is indicated by the color bar. Corresponding 2D projections of the Pt atomic density onto the XY, XZ, and YZ planes are also presented, highlighting areas of higher Pt concentration. This is an interactive plot, hence one can scroll the axis, zoom in/out to probe the regions of interest.

The volumetric density maps were generated for the Pt_1_, Pt_3_, and Pt_5_ embedded in CHA zeolite system for simulations run at 750, 1000, and 1250 K (see Figures S9, S11, S13), as well as their 2D density projections (see Figures S10, S12, S14). They reveal distinct spatial behaviors depending on the size of the platinum cluster. As can be seen below from Figure 10 for Pt_5_ in the CHA zeolite system at 1250 K.

The Pt_5_ cluster generally shows strong confinement within the CHA cages, with sharp localization at cage centers and no detectable density outside the cage (see Figures S13 and S14). This stays the same even at elevated temperatures such as 1250 K in Figure 10. The corresponding 2D projections further confirm this strong confinement.

For completeness, we briefly summarize the behavior of Pt_1_ and Pt_3_ clusters (see Table S4 and Figures S9 and S11 and their 2D density projections in Figures S10 and S12). Pt_1_ remains strongly localized near 6‐membered rings across all temperatures, with only minor increases in hopping between adjacent sites at higher temperatures. Pt_3_ displays a more diffuse density distribution and increased thermal motion, occupying larger cages and exhibiting transient migration attempts at elevated temperatures. These findings are consistent with previous interpretations and further validate the tool's ability to capture spatial dynamics. Full results and detailed analysis for Pt_1_ and Pt_3_ are provided in Supporting Information: Section 3.3.2.

### 3.4 Case Study: Bulk Water System

In the sections that follow, we showcase the robustness and versatility of the CRISP framework by re‐analysing ab initio molecular‐dynamics trajectories of liquid water originally reported by Villard et al. [47]. In their work, all simulations employed a cubic box of volume $\left(12.4453^{3}\;{Å}^{3}\right)$ containing 64 $D_{2}O$ molecules ($\rho \approx 1\;g\;{\mathrm{cm}}^{-3}$), propagated via Car–Parrinello MD with a time steps of 4 a.u. (≈0.097fs). Starting from a pre‐equilibrated classical snapshot, each functional (M06‐L, M11‐L, MN12‐L, MN15‐L, revM06‐L) underwent heating to 400 K over 1 ps via velocity rescaling, then cooling to 330 K over 1 ps, then to 300 K over 0.5 ps. Ultimately, NVT thermalization at 300K for several picoseconds using a Nosé–Hoover thermostat was carried out, followed by production runs in the NVE ensemble for at least 10 ps, saving configurations every 50 steps.

#### 3.4.1 Mean–Square Displacement and Diffusion Coefficients

**FIGURE 11:**
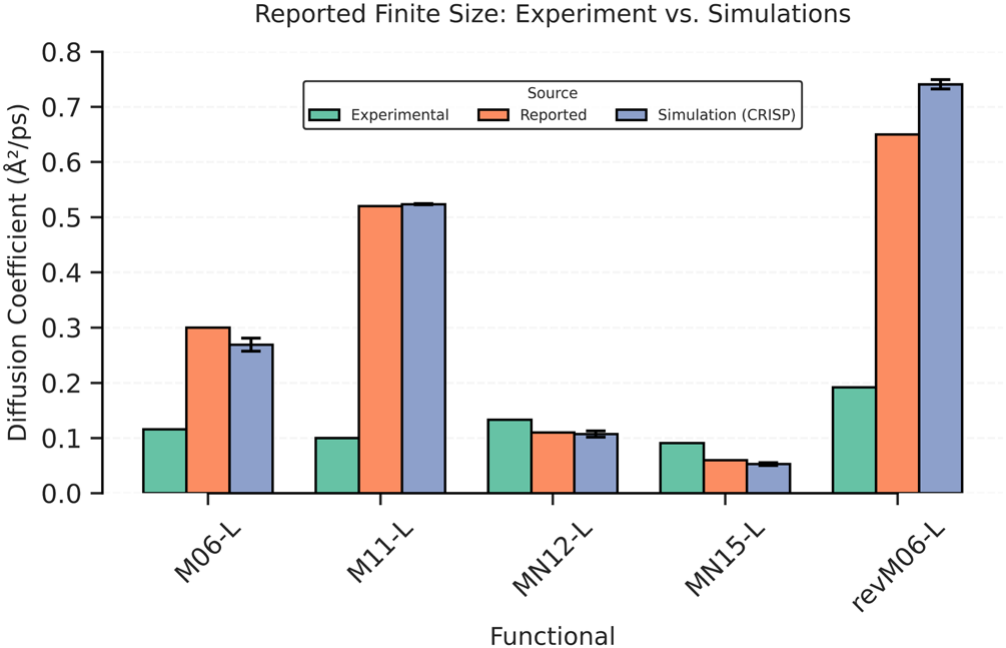
Comparison of water finite‐size self‐diffusion coefficients (in ${Å}^{2}/\mathrm{ps}$) across five meta‐GGA density functionals. Experimental benchmarks as per Villard's work [47] are represented by the green bars. The orange bars represent diffusion coefficients from the original simulations reported by Villard et al. (no error bars are provided for these values, as they were not given in the paper), and the blue bars show results obtained using the CRISP analysis module, which includes error bars reflecting statistical uncertainty.

To benchmark CRISP's capabilities for dynamical property extraction, we applied its mean square displacement (MSD) analysis module to evaluate water‐oxygen diffusion in trajectories generated with different meta‐GGA DFT functionals. CRISP supports both single‐origin and windowed (multi time‐origin) MSD calculations (see Supporting Information: Section 2.4), and all results presented here use the more robust windowed approach. Figure 11 compares three sets of self‐diffusion coefficients: experimental values at 298 K, diffusion coefficients originally reported by Villard et al. [47], and those computed here using CRISP. We focus exclusively on finite‐size diffusion coefficients, as the correction for the infinite‐size limit simply adds the same value to both CRISP and VMD results. Therefore, the comparison between methods shall remain unchanged, and details on the correction, infinite‐size values are provided in the original work by Villard [47].

Villard et al. used the Diffusion Coefficient Tool plugin for VMD [48, 49] to calculate the self‐diffusion coefficient from the mean square displacement (MSD) using the Einstein relation (Supporting Information: Equation 13). CRISP and the VMD Diffusion Coefficient Tool [48, 49] both: (i) compute a time–origin averaged MSD ($M\left(t\right)$) using a multiple time–origin (“window”) scheme; and (ii) estimate (D) from a linear relationship ($M\left(t\right)\approx 2\mathrm{dDt}+b$, where Dimension d=3) over a user–selected diffusive interval/zone. While the VMD tool uses a single global linear fit over the chosen interval, CRISP uses a block‐averaged mode that splits the interval into user‐defined subwindows, performs independent linear fits in each, and reports the mean (D) together with its standard error of the mean (SEM). The predicted diffusion coefficients from these approaches, as well as experimental values, are reported in Figure 11. There are only minor deviations between the two theoretical predictions, which most likely arise from minor differences between CRISP and the VMD Diffusion Coefficient Tool in MSD fitting, lag time selection, uncertainty estimation, and so on, with Villard et al. not reporting some important details of their specific set‐up, such as the specific fitting intervals, and lag times used for the diffusion coefficient evaluation. For the CRISP analysis presented here, we rely on a standard practice to fit the linear regime of the MSD data using all available lag times. Detailed descriptions of these methodological distinctions, along with a tabulated validation of diffusion coefficients, are provided in Supporting Information: Section 3.4.1 and Table S7.

Based on a direct comparison presented in Figure 11, several clear patterns emerge from our analysis of both tools:

- *Results for the functionals*: For all functionals, the diffusion coefficients computed by CRISP match those reported using the VMD method reasonably well. However, some mismatches are observed, particularly for M06‐L and revM06‐L, where CRISP yields slightly lower and higher values, respectively, compared to the reported results. Overall, the similarity between CRISP and VMD results across all functionals demonstrates that both analysis approaches yield consistent diffusion estimates from the same simulation data, with a few cases of small variations.
- Experiment vs. both methods: As shown in the plot, both CRISP and the VMD method deliver similar accuracy with respect to the experimental diffusion coefficient for all functionals. The largest discrepancies are observed for M11‐L and revM06‐L. For MN12‐L and MN15‐L, the simulation results are closer to the experiment. The discrepancies between simulation and experiment are primarily due to differences in the level of theory, simulation temperature, timestep, and trajectory length, rather than any inherent limitation of the CRISP or VMD analysis approaches.

For detailed illustration of the analysis workflow and fitting procedures used by both methods, see Figures S15 and S16. These figures highlight the methodological similarities and differences in implementation (graphical user interface in VMD versus Python scripting in CRISP) and other necessary details.

Overall, both tools apply the same physical principles to estimate diffusion coefficients, but differ in their fitting and averaging strategies. CRISP's block‐averaged fitting and explicit uncertainty evaluation offer a systematic approach for quantitative analysis, even for longer simulation trajectories with exhaustive and flexible settings, while the VMD tool remains convenient for rapid, visual exploration, often aiming for default (less flexible) settings or smaller trajectories.

#### 3.4.2 Radial Distribution Function

**FIGURE 12:**
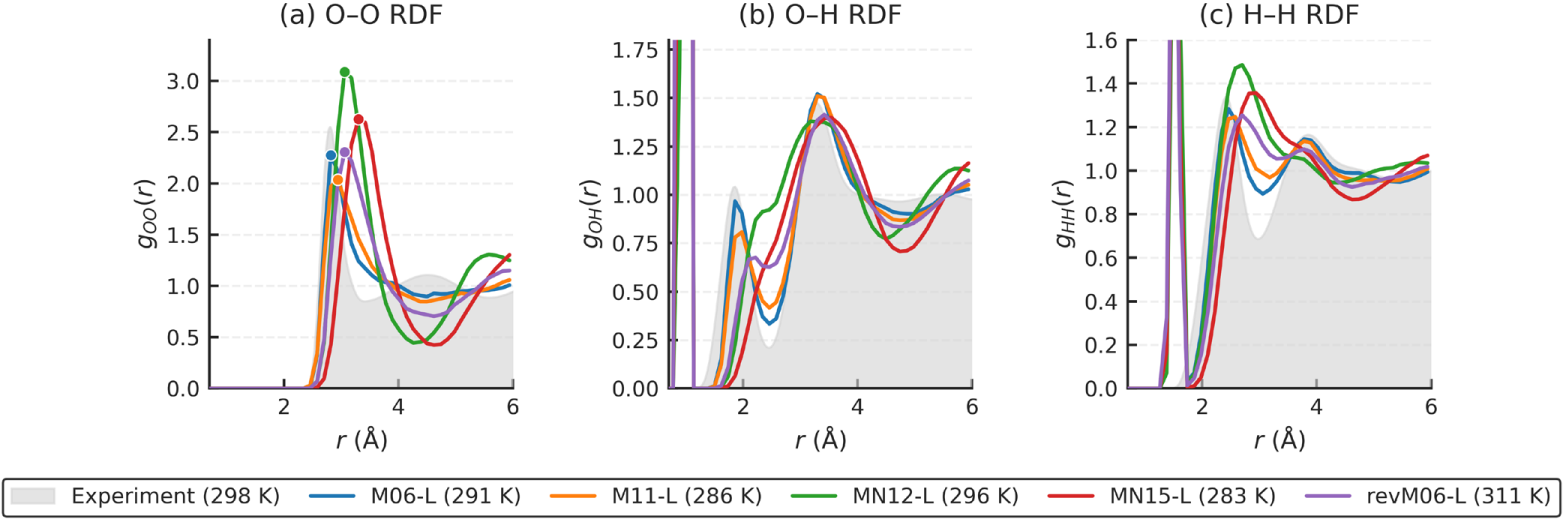
Oxygen–Oxygen (O—O), Oxygen–Hydrogen (O—H), and Hydrogen–Hydrogen (H—H) radial distribution functions (RDFs) for water computed using the CRISP software. The RDFs are calculated on the ab initio molecular dynamics trajectories originally provided by Villard et al. [47] using various meta‐GGA functionals (M06‐L, M11‐L, MN12‐L, MN15‐L, revM06‐L). Experimental RDF data at 298 K are shown in grey for comparison.

Radial Distribution Functions are a standard metric that represents the structural correlations in molecular simulation data such as AIMD trajectories. Here, using the trajectories from the original work [47], we computed the RDFs for all three element pairs (O—O, O—H, H—H) with CRISP. Our CRISP results are presented in Figure 12, alongside experimental reference at 298 K provided in the original work [47]. The $g_{\mathrm{OO}}$ is obtained from x‐ray diffraction, for $g_{\mathrm{OH}}$ and $g_{\mathrm{HH}}$, the results are from interpolated joint X‐ray/neutron diffraction experiments, all at 298 K.

**FIGURE 13:**
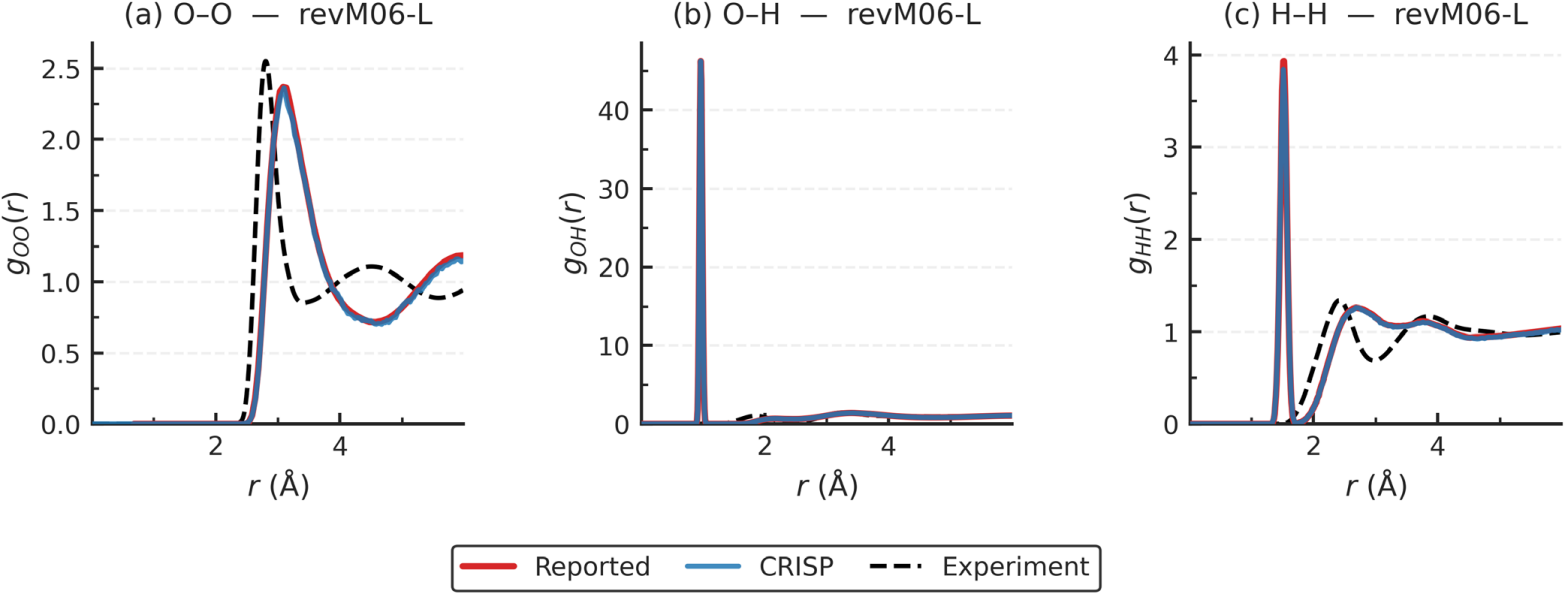
A comparison of all radial distribution functions (RDFs) for water, calculated for the revM06‐L meta‐GGA functional. The RDFs were computed using ab initio molecular dynamics trajectories from Villard et al. [47]. The RDF obtained using the CRISP software is shown in blue. The RDFs from Villard et al. are shown in red.

The calculation of RDFs from molecular simulations involves specific algorithmic choices (binning, averaging across trajectory frames, and normalization procedure, etc.) that can influence the final output. Villard et al. computed RDFs using the VMD software [48]. The CRISP, through the dedicated modules, used the same binning as reported by Villard et al. [47]. All RDFs including experimental are provide in Figure 13 (also provided in Table S6), with the several trends emerging:

- O—O RDFs: As shown in Figure 13, the CRISP results closely match the reported data for the O–O pair (homo‐atoms) across all functionals (see Figure S17), with both deviating similarly from the experimental values. This agreement is quantitatively shown by the Mean Absolute Errors (MAEs) in Table S5, where the MAE values for CRISP and the reported method are nearly identical (0.2928 vs. 0.2961), confirming that the analysis method has minimal impact on the results.
- O—H RDFs: The O—H RDFs (hetero‐atoms) also show strong agreement between CRISP and the reported method, as seen in Figure 13 and Figure S18. The MAEs in Table S5 are identical (0.5594 for both), further demonstrating that any differences from experiment arise from the underlying simulation and not the analysis approach.
- H—H RDFs: For H—H RDFs (homo‐atoms), Figure 13 and Figure S19 illustrate that both CRISP and the reported method produce nearly overlapping curves, with only minor deviations from experiment. The MAEs in Table S5 are again very close (0.1947 for CRISP vs. 0.1954 for reported), reinforcing the consistency between the two analysis methods.

Hence, any differences observed between the computed RDFs and experimental data for both CRISP and the reported VMD method arise from the nature of the simulation trajectories and the underlying theoretical models, rather than from the analysis methods themselves.

Finally, in Supporting Information, we also conducted a comparison of the CRISP and VMD approaches to a standard RDF implementation in the ASE. The aim was to highlight the differences in normalization, calculation methods and their impact on the RDFs (see Figures S15, S16, and S20 related discussion).

A comparative advantage of CRISP over other methods available is that it handles custom index‐based calculations, going beyond the atom types (oxygen, hydrogen, etc.). Hence, one can calculate the RDF for a subset of atom types to probe the specific behavior of the atoms of interest. Also, the results are accompanied by the animated frame‐wise RDF plot that provides information on the evolution of atomic arrangements throughout the trajectory.

#### 3.4.3 Coordination Number

**FIGURE 14:**
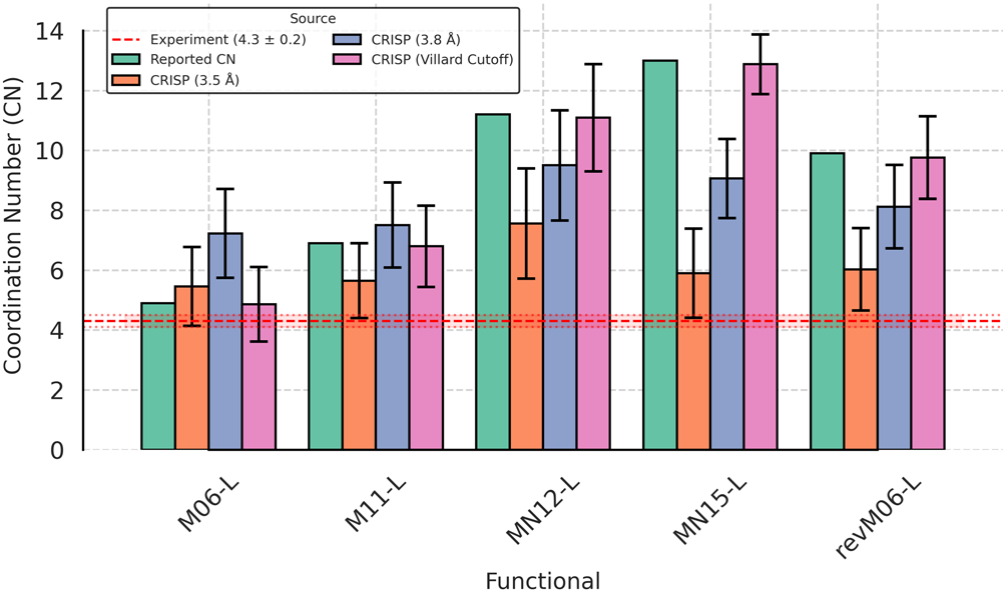
Comparison of Oxygen–Oxygen coordination numbers (CN) for water calculated using different meta‐GGA functionals. Green bars represent the CN values reported by Villard et al. [47], derived from the first minimum of the O‐O RDF. Orange and blue bars show the CN calculated by CRISP using fixed cutoff distances of 3.5 and 3.8 Å, respectively. The purple bars show the CN calculated using a dynamic cutoff approach, as employed by Villard et al. The dashed red line indicates the experimental CN value of 4.3 ± 0.2.

Herein, we focused on obtaining the coordination number (CN) of a water molecule (specifically, the oxygen atom of water) with other water molecules (i.e., other oxygen atoms) in its first solvation shell. Figure 14 compares CN values derived using two methodologies: (1) the original approach by Villard et al., which integrates the O—O RDF up to its first minimum (dynamic cutoff), and (2) CRISP's fixed‐distance cutoffs (3.5 Å and 3.8 Å). The 3.5 Å cutoff aligns with the experimental first coordination shell (2.8–3.4 Å), while 3.8 Å extends beyond it, capturing more neighbors. The insights from the results of the two methods are as follows:

- *Flexibility and interpretability*: By allowing the user to define fixed cutoffs (e.g., 3.5 Å or 3.8 Å), CRISP provides flexibility to investigate coordination number using the same set‐up, which can be useful when well‐defined features (minima, maxima) in RDF are not clearly discernible.
- *Functional‐specific trends*: MN12‐L and MN15‐L, which overstructure water, exhibit higher CN values even at 3.5 Å (5.2 and 5.4, respectively), consistent with their exaggerated O—O RDF peaks. M06‐L shows the closest agreement with experiment (CN = 4.6 at 3.5 Å), reflecting its intermediate structuring.
- *CRISP with Villard cutoff*: We also used the dynamic cutoffs (i.e., determined by the first minimum of the O—O RDF) to compare the consistency of CRISP implementation with respect to the work of Villard et al. (Figure 14). It confirms that the results obtained by the CRISP prdf module closely follow those of Villard et al., as can be seen from the MAE value = 0.10 between the two methods (see Supporting Information: Section 3.4.3 and Table S8 for specific functional breakdowns).

#### 3.4.4 Hydrogen Bond

**FIGURE 15:**
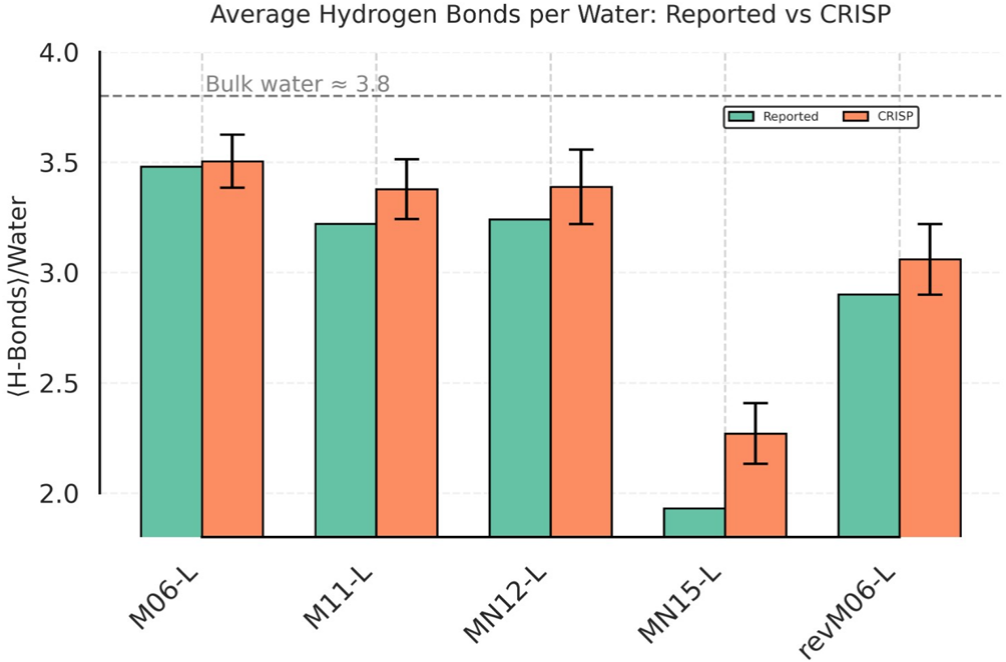
Comparison of the average number of hydrogen bonds per water molecule for bulk water AIMD trajectories obtained using different meta‐GGA functionals. Green bars show the values reported by Villard et al. [47], derived from O‐O RDF analysis. Orange bars represent the values calculated by CRISP using geometric criteria based on angle and distance cutoffs involving oxygen atoms (see Section 2.2). Error bars for CRISP data indicate the standard deviation across trajectory frames. The dashed grey line represents the approximate experimental value for bulk water (≈3.8).

Finally, we examine the hydrogen‐bonding network, a critical aspect of water's structure and dynamics. We compare in Figure 15 the average number of hydrogen bonds (H‐bonds) per water molecule calculated by CRISP with the values reported by Villard et al., also reporting the approximate experimental value for bulk water.

The Villard et al. [47] derived H‐bond information from the oxygen–oxygen radial distribution function (O–O RDF). Instead of strict cutoffs, they employed smoothed functions to describe the probability of an H‐bond based on two geometric parameters (oxygen–oxygen distance and donor‐hydrogen‐acceptor distance) and an H‐bond was identified if the product of these two functions exceeded a specific threshold of 0.5 (see Supporting Information: Section 3.4.4 for more details and Table S9 for a numerical comparison of average H‐bond counts across all functionals).

CRISP employs a well‐established criterion [50] based on donor‐hydrogen‐acceptor angle and hydrogen‐acceptor distance cutoffs (specifically, angle and distance criteria, as detailed in Section 2.2). This direct geometric definition is commonly used in H‐bond analysis and provides a clear and reproducible measure of local connectivity.

The comparison in Figure 15 shows that both methods capture the same trends for all meta‐GGA functionals, indicating that despite their different parameterizations, they largely agree on the relative hydrogen bonding propensities predicted by each functional. Notably, the CRISP approach reports mildly larger H‐bond networking across the board (which happens to agree better with the experimental observations).

Overall, through these case studies (Sections 3.4.1, 3.4.4), we attempted to demonstrate CRISP's utility in extracting reliable structural metrics from (AIMD) trajectories, offering various utilities that make it a robust and flexible platform. This flexibility can be used to extract important details about the specific systems in question.

## 4 Conclusions

In this work, we introduced CRISP as a post‐processing tool for molecular simulations that attempts to provide interactivity, efficient handling of large datasets, and flexibility in workflow customization. By integrating seamlessly with the Atomic Simulation Environment (ASE) and leveraging Python's robust scientific ecosystem, CRISP provides a modular, open‐source platform that enhances the analytical capabilities for researchers in materials science and chemistry. Its key features, such as parallel processing for high‐performance computing, usage of advanced algorithms like DBSCAN clustering and SOAP‐based subsampling, and interactive 3D visualizations, enable efficient extraction of atomic‐scale insights from complex simulation data.

The software's consistent API design, extensive documentation, and compatibility with common data formats further lower the barrier to adoption, making it easier for researchers to integrate CRISP into their existing methods for analysis.

The case studies presented in this work demonstrate CRISP's versatility and accuracy across diverse applications, from analysing platinum cluster dynamics in zeolites to quantifying water diffusion and hydrogen‐bonding networks using ab initio trajectories. Hence, CRISP provides a platform for generating reproducible workflows and reduces the time researchers need to spend on code development, allowing them to make use of this existing codebase to focus on scientific discovery.

## Funding

The authors acknowledge the support of the Czech Science Foundation (23‐07616S). This work was supported by the Ministry of Education, Youth and Sports of the Czech Republic through the e‐INFRA CZ (ID:90254). In addition, Charles University Centre of Advanced Materials (CUCAM) (OP VVV Excellent Research Teams, project number CZ.02.1.01/0.0/0.0/15_003/0000417) is acknowledged.

## Conflicts of Interest

The authors declare no conflicts of interest.

## Supporting information


**Data S1:** jcc70384‐sup‐0001‐Supinfo01.pdf.

**Data S2:** jcc70384‐sup‐0002‐Supinfo02.pdf.

## Data Availability

The data that supports the findings of this study are available in Supporting Information of this article.
