# Supplementary material for: CRISP: Enhancing ASE Workflows With Advanced Molecular Simulation Post‐Processing
Source: J Comput Chem. 2026 May 6;47:e70384. doi: 10.1002/jcc.70384 (PMC13150413; doi:10.1002/jcc.70384)
Supplement: Supplementary file 1 — Data S1: jcc70384‐sup‐0001‐Supinfo01.pdf. [file JCC-47-0-s002.pdf]

SOFTWARE NEWS AND UPDATES
Journal Section

CRISP: Enhancing ASE Workflows with Advanced Molecular Simulation Post-Processing

Indranil Saha[1*] | Daniel Willimetz[1†] | Lukáš Grajciar*[1‡]

1 | SCIENTIFIC JUSTIFICATION AND COMPARATIVE MAPPING

The design philosophy of CRISP (Comprehensive Repository for Insightful Simulation Postprocessing) is to function as a robust bridge between high-volume molecular simulation outputs and the extraction of actionable scientific insights. The architecture specifically addresses the requirements of the materials science community by providing researchers with a modular platform for turnkey and reproducible data analysis. This framework is particularly optimised for machine learning practitioners who utilise descriptors like Smooth Overlap of Atomic Positions (SOAP) for dataset subsampling as well as High Performance Computing (HPC) users who require parallelised processing to handle voluminous trajectories efficiently.

The following section provides a comparative mapping of these capabilities against established molecular analysis platforms to highlight the specific technical advancements and scientific innovations introduced by the CRISP toolkit.

1.1 | CRISP vs. Existing Molecular Analysis Platforms

We present a detailed technical comparison between CRISP and five widely used platforms: PyMol, VMD, OVITO, MDAnalysis and native ASE modules. This comparison highlights CRISP's strengths in integrated analysis workflows with emphasis on error propagation capabilities, parallel processing, interactive volumetric analysis and clustering-driven structure discovery.

1.2 | Key Differentiators

CRISP introduces specific capabilities that systematically distinguish it from existing platforms, as detailed in Table 1.

1. Integrated Error Analysis Pipeline

CRISP uniquely provides native error propagation through `error_analysis.py`, implementing autocorrelation function (ACF) and block averaging—capabilities absent in PyMol, VMD, OVITO, MDAnalysis, and ASE. Key functions:

- `autocorrelation_function()`: Computes normalised ACF with FFT optimisation (O(N log N))
- `integrated_autocorrelation_time()`: Estimates τ_{int} via windowed summation (Sokal criterion: $\tau_{\text{max}} = 5\tau_{\text{int}}$)
- `block_average()`: Performs convergence-tested block averaging with configurable block sizes
- `plot_convergence()`: Generates dual-panel convergence plots (ACF decay, block size vs. standard error)

Competing platforms require manual uncertainty propagation via external packages (statsmodels, scipy). CRISP's integrated approach ensures:

- Automatic convergence detection ($T \geq 20\tau_{\text{int}}$ criterion) via `check_convergence()`
- Reliable standard error estimates accounting for time correlations
- Reproducible uncertainty reporting across MSD (`msd.py`), RDF (`prdf.py`), and all statistical modules
- Automated export of uncertainty data

Validation Evidence: In GitHub, the `Specific_Tutorial/Error_Analysis/` tutorial (`example.py`) demonstrates convergence analysis for ^{27}Al NMR chemical shifts in zeolite MFI, showing reduction of standard error from 64.3 kJ/mol (1 ps) to 0.8 kJ/mol (1 ns) via both ACF ($\tau_{\text{int}} = 50$ fs) and block averaging. Output files (`Al_nmr_results.txt`, `energy.npy`)

are provided in example/Specific_Tutorial/Error_Analysis/.

2. Machine Learning-Native Dataset Subsampling via SOAP-Farthest Point Sampling

CRISP provides a dedicated machine learning pipeline through subsampling.py, enabling efficient construction of the training set for machine learning interatomic potentials (MACE, SchNet, NequIP). Key capabilities:

- compute_soap_descriptors(): Computes rotation-invariant SOAP descriptors via DSCRIBE with configurable radial/angular bases, Gaussian width sigma, exponent ζ, and species weighting—functionality absent in PyMol, VMD, OVITO, MDAnalysis, and ASE
- farthest_point_sampling(): Greedy FPS over SOAP space to select maximally diverse frames (approx. $O(N^2)$)
- write_subsampled_trajectory(): Exports subsampled structures for direct use in MACE/SchNet/NequIP
- compute_soap_distance_matrix(): Optional distance precomputation for FPS convergence monitoring

Quantitative Validation (rMD17): Notably, the model trained on only 1000 FPS selected structures reached a 10× reduction in dataset size while achieving better energy accuracy and comparable force accuracy than the 10000 structure random set. Tutorial code (example.py) and outputs (subsample_rMD17.xyz) are provided in the GitHub example/Specific_Tutorial/Subsampling/.
Complete workflow demonstrated in example/Workflow/collage_calculations.ipynb.

3. Optimised Interactive Volumetric Probing

The volumetric_atomic_density.py module provides real-time, interactive 3D atomic density mapping via Plotly:

- compute_volumetric_density(): 3D histogram with PBC handling
- create_density_plot(): Isosurface rendering with atomic species colouring, opacity, and threshold control
- add_2d_projections(): XY/XZ/YZ projections via NumPy summation with contour overlays—enabling simultaneous 3D and planar density analysis
- save_density_data(): NPZ export (nvt_density_data.npz) with grid metadata for reproducibility

While PyMol, VMD, and OVITO offer volumetric visualisation, CRISP uniquely exports standalone HTML files (SiAl15_density_no_projections.html, SiAl15_density_with_projections.html) with interactive controls (zoom, rotation, species filtering) without requiring GUI tools or expensive licenses (OVITO Pro). Tutorial outputs (Images shown) available in GitHub example/Introductory_Tutorial/SiAl15_Density/.

4. Advanced Trajectory Visualisation and Contact Analysis

CRISP provides native interactive 3D trajectory rendering and comprehensive contact/coordination quantification.

Atomic Trajectory Line Mapping (atomic_traj_linemap.py**:** Renders interactive 3D trajectories as continuous lines or point clouds with frame-by-frame annotations and standalone HTML export. Other tools require custom scripting or plugins for equivalent functionality.
Tutorial outputs in GitHub: example/Introductory_Tutorial/atomic_traj_linemap/

Contact-Coordination Analysis (contact_coordination.py**:** Generates interactive contact-time heatmaps with res-

olution and distance matrices with species-resolved atom labels. Provides time-series coordination number analysis with distribution histograms and per-frame statistics. VMD requires TCL scripting; MDAnalysis requires custom code; PyMol/OVITO/ASE offer limited functionality.
Tutorial outputs in GitHub: `example/Introductory_Tutorial/CN_data/` and `Contacts_data/`.

5. Time-Resolved RDF Animation and H-Bond Network Analysis

CRISP provides dynamic RDF animations and NetworkX-based hydrogen-bond network analysis with interactive Plotly visualisations.

Dynamic RDF (`prdf.py`): Computes total/partial RDF per frame and exports dynamic animations showing structural evolution over time. VMD, OVITO, and MDAnalysis require custom scripting; PyMol lacks this capability; ASE's RDF implementation contains bugs for heteroatom pairs (SI Figure 20).
Tutorial code in GitHub: `example/Specific_Tutorial/RDF/example.py`.

H-Bond Network Graphs (`h_bond.py`): Builds per-frame and time-averaged hydrogen-bond networks using NetworkX, rendering interactive Plotly HTML with edge distances, node degree/size, and correlation-matrix heatmaps with hover tooltips. Exports donor-acceptor indices and per-frame H-bond counts. VMD HBonds plugin and MDAnalysis require manual post-processing; PyMol/OVITO/ASE lack network graph capabilities.
Tutorial code in GitHub: `example/Specific_Tutorial/H_Bond/example.py`.

6. Directional MSD with Windowed Ensemble Averaging

`msd.py` implements windowed ensemble averaging over all time origins for robust MSD statistics, plus directional diffusion analysis (X/Y/Z components) with block-averaging error estimation. Key advantages:

- **Windowed ensemble averaging**: Improves statistical convergence by computing MSD from multiple time origins (unlike single-origin VMD plugin or ASE limited implementation)
- **Directional MSD**: Useful for identifying anisotropic diffusion (say in confined channels in zeolites)—unavailable in PyMol, VMD, OVITO, and limited in MDAnalysis/ASE
- **Block-averaging integration**: Automatic uncertainty quantification via `error_analysis.py`
- **CSV export**: `msd_results.csv` with time, MSD, and directional components

Tutorial outputs in GitHub: `example/Introductory_Tutorial/traj_csv_detailed/msd_results.csv`.

7. Clustering-Driven Structural Discovery

CRISP enables trajectory clustering for structural motif discovery and dataset reduction via scikit-learn integration.

Frame-Level Clustering (`clustering.py`, `example_frame.py`): Generates interactive HTML scatter plots showing spatial distribution of clusters with frame-by-frame metadata.

Trajectory-Level Clustering (`example_traj.py`): Produces first-frame and last-frame visualisations plus CSV exports usable for downstream ML stratification and active learning.

ML Integration: Wraps DBSCAN algorithms; can be integrated with `atomic_indices.py` for species-filtered cluster-

ing and `subsampling.py` to prioritise diverse clusters in FPS. VMD and OVITO offer limited clustering (manual setup); MDAnalysis requires external scikit-learn coding; PyMol and ASE lack clustering tools.
Tutorial code in GitHub: `example/Specific_Tutorial/Clustering/example_frame.py` and `example_traj.py`.

8. Unified Atomic Indexing and Distance Calculations

CRISP provides consistent atomic identification and vectorised distance computation across all modules.

Atomic Indexing (`atomic_indices.py`): Symbol-based atomic selection with automated NPY export (`Al/H/O/Si_indices.npy`) and species-specific cutoff estimation (`cutoff/*.csv`). Ensures consistent atom mapping across all 11 modules.
Tutorial outputs in GitHub: `example/Introductory_Tutorial/indices_new/`.

Interatomic Distances (`interatomic_distances.py`): Computes vectorised pairwise distances with caching for reuse in RDF, clustering, and ML feature extraction.
Tutorial outputs in GitHub: `example/Introductory_Tutorial/distance_calculations_zeo/`.

9. Modular Analysis Composition with Consistent API

All 11 modules (`data_analysis/`: 6 modules; `simulation_utility/`: 5 modules) share:

- **Standardised parameters**: `traj_path`, `frame_skip`, `output_dir`, `atom_indices`
- **Consistent units**: femtoseconds (time), ångströms (distance), degrees (angles)
- **Automated outputs**: CSV (tabular data), NPY (arrays), HTML (interactive Plotly visualisations)
- **Native parallelisation**: Joblib-based multi-core processing without external Dask/TCL scripting (unlike MDAnalysis/VMD)

Workflow Efficiency Evidence: In the GitHub, there is example workflow, `example/Introductory_Tutorial/CRISP_Introductory_Tutorial.ipynb` (FAU zeolite + water) it executes RDF, MSD, H-bonding, clustering, volumetric density, contact-coordination, and trajectory visualisation analyses in a single Jupyter notebook, producing organised HTML/CSV/NPY outputs across subdirectories without format conversion or manual data reshaping.

TABLE 1 Technical Feature Comparison Matrix: CRISP vs. PyMol, VMD, OVITO, MDAnalysis and ASE (native)

Feature Category	CRISP	PyMol	VMD	OVITO	MDAnalysis	ASE
Data Handling						
Native atomic indexing system	✓✓	✓	Limited	Limited	✓	Limited
Built in parallelisation	✓✓	✓	✓(scripting)	✓	✓(Dask Library)	Limited
Machine Learning Integration						
SOAP descriptor computation	✓✓[a]	✓	✓	✓	✓	✓
Farthest Point Sampling (FPS)	✓✓[a]	✓	✓	✓	✓	✓
Dataset subsampling for ML	✓✓	✓	✓	✓	✓	✓
Volumetric and Spatial Analysis						
Atom trajectory line/point 3D	✓✓[b]	Limited	Limited (plugin)	✓	Limited (custom)	Limited (custom)
Atomic density mapping	✓✓[c]	Limited	✓	✓✓	✓	✓
Time-resolved RDF animation	✓✓[d]	✓	Limited (scripting)	Limited (pipeline)	Limited (custom)	Limited (Buggy)
Atom contact-time heatmaps	✓✓[e]	Limited	✓(TCL[k])	Limited	✓	Limited
Detailed atom coordination	✓✓[e]	Limited	✓(TCL[k])	Limited	✓	Limited
Statistical Analysis						
Detailed MSD (windowed ensemble, error estimation)	✓✓[f]	✓	✓[g]	✓	✓	Limited
Directional MSD (X/Y/Z components)	✓✓[f]	Limited	Limited	Limited	Limited	Limited
Clustering algorithms	✓✓	✓	✓	✓(limited)	✓(scripting)[h]	✓
Hydrogen bonding analysis	✓✓	✓	✓[i]	Limited	✓	✓
Interactive H-bond network graph	✓✓[j]	✓	Limited (plugin)	Limited	Limited (custom)	✓
H-bond atom correlation matrix	✓✓[j]	✓	Limited (plugin)	Limited	Limited (custom)	✓
Statistical error analysis	✓✓[l]	Limited	Limited	Limited	Limited (custom)	Limited
Workflow Integration						
Chained analysis operations	✓✓	Limited	Limited (TCL[k])	Limited	✓	Limited
Consistent API	✓✓	✓	Limited (TCL[k])	Limited	Limited	Limited
Programmatic Python interface	✓✓	✓	Limited	✓	✓✓	✓✓
Open source licence	✓✓	✓	Limited[m]	✓	✓✓	✓✓

[a] CRISP `subsampling.py` computes SOAP descriptors (DScribe) and applies FPS to subsample trajectories for ML workflows.
[b] CRISP `atomic_traj_linemap.py` renders interactive 3D trajectories (lines or point clouds) with annotations and HTML export.
[c] CRISP `volumetric_atomic_density.py` generates interactive 3D atom-density isosurfaces, XY/YZ/XZ projections, and saves NPZ grids/edges.
[d] CRISP `prdf.py` computes total/partial RDF per frame and exports dynamic animations (HTML/GIF).
[e] CRISP `contact_coordination.py` renders interactive contact-time heatmaps (ps) and distance matrices (min/mean/std), and provides time-series coordination analysis with CN distributions and uncertainty.
[f] CRISP `msd.py` implements windowed ensemble averaging over all time origins for robust MSD statistics and directional diffusion analysis, with block-averaging error estimation.
[j] CRISP `h_bond.py` builds per-frame and averaged hydrogen-bond networks with NetworkX and renders interactive Plotly HTML (edge distances, node degree/size), plus interactive correlation-matrix heatmaps with hover tooltips.
[l] CRISP `error_analysis.py` estimates statistical errors via ACF and block averaging, with plots.
[g] VMD Diffusion Coefficient Tool plugin; [h] requires external scikit-learn; [i] HBonds plugin; [m] source available, but compilation is not user-friendly. [k] TCL = Tool Command Language (scripting language used by VMD).

1.3 | Flexibility of CRISP

A key architectural principle underlying CRISP is its modular design philosophy, which enables researchers to seamlessly extend the toolkit with custom analyses without modifying core library code. This flexibility is demonstrated through a practical tutorial: Bond-Angle Distribution (BAD) analysis for Si–O–Si triplets in aluminosilicate frameworks. This use case exemplifies how domain-specific structural insights can be extracted by composing existing CRISP utilities with user-defined analysis logic.

The Si–O–Si bond angle is a critical descriptor of zeolite framework geometry, with well-documented variability in the literature. Experimental and computational studies show:

- **Aluminosilicate Zeolites**: Presence of Al substitution reduces flexibility, with mean angles around 142° for Si–O–Si [1]

CRISP enables rapid prototyping of such structural metrics by reusing atomic indexing, trajectory I/O, and output conventions. The BAD tutorial (`example/Specific_Tutorial/Bond_Angle/`) demonstrates this.

Step 1: Atomic Indexing
Load atomic indices using CRISP's standardised pattern:

```
si_indices = load_atomic_indices_from_file(indices_dir, "Si")
o_indices  = load_atomic_indices_from_file(indices_dir, "O")
```

Step 2: Geometric Feature Identification
Identify Si–O–Si triplets using cutoff distance (2.2 Å):

```
triplets = find_bridging_oxygen_triplets(
    positions, si_indices, o_indices, cutoff=2.2
)
```

Step 3: Frame-by-Frame Analysis
Compute angles over trajectory with optional subsampling:

```
angle_data = analyze_bond_angles(traj_file, triplets, skip=10)
```

Step 4: Multi-Format Output
Generate standardised outputs (CSV, TXT, PNG, HTML):

```
write_angles_to_csv(...)              # Raw data
write_histogram_to_csv(...)           # Distribution
write_statistics_to_txt(...)          # Summary statistics
create_distribution_plot_matplotlib(...) # Publication-ready PNG
create_distribution_plot_plotly(...)     # Interactive HTML
```

This four-step pattern mirrors CRISP's established conventions in `h_bond.py`, `msd.py`, and `contact_coordination.py`, enabling rapid extension without modifying core code.

Tutorial outputs (`example/Specific_Tutorial/Bond_Angle/BAD_data/`) demonstrate quantitative agreement with literature Si–O–Si angle distributions, validating the newly added CRISP codes.

The BAD analysis outputs (see Figure 1) demonstrate quantitative agreement with published experimental and computational data. Tutorial results from the FAU zeolite + water system show:

- **Mean Si–O–Si Angle**: 153.4° ± 8.2°
- **Distribution Range**: 127° to 179° (matching literature ranges zeolite frameworks [1, 2])

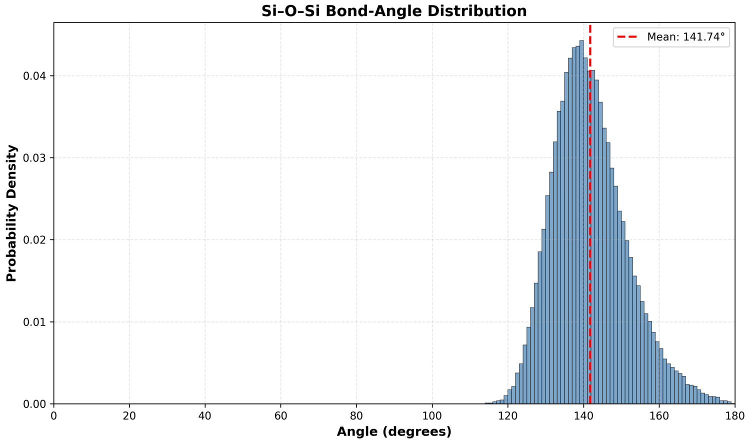

FIGURE 1 CRISP Flexibility: Si–O–Si bond-angle distribution for the FAU aluminosilicate system. The histogram (normalised probability density) shows angles computed over the trajectory, with a mean of 141.7° (red dashed line). The distribution spans ∼ 120°–170°, consistent with reported flexibility ranges in aluminosilicate zeolites.

1.4 | Detailed Comparison Against Specific Platforms

PyMol vs. CRISP

PyMol excels as a static visualisation tool for protein structures, but lacks:

- Programmatic batch analysis (GUI-centric; Python API limited to rendering)
- Trajectory statistics (no MSD, RDF, H-bond time series)
- Error quantification (no ACF or block averaging)
- Parallelisation and modular composition
- ML integration (no SOAP/FPS capabilities)

CRISP Advantage: Python-native interface with NumPy/SciPy/scikit-learn integration, producing standalone HTML visualisations without GUI dependencies.

VMD vs. CRISP

VMD is widely used for trajectory visualisation, but has notable limitations:

- TCL scripting (steep learning curve; Python wrapper limited)
- Plugin fragmentation (inconsistent output formats across HBonds, MSD, RDF plugins)
- Manual error propagation (no integrated τ_{int} estimation or block averaging)
- Closed ecosystem (GPL source available but compilation complex)
- GPU acceleration rendering-only (analysis remains CPU-bound)

CRISP Advantage: Native Python interface eliminates TCL scripting with integrated error propagation and interactive contact-coordination heatmaps unavailable in default plugins.

OVITO vs. CRISP

OVITO provides GPU-accelerated visualisation, but:

- Python API only in Pro version (commercial license required)
- Modifier pipeline architecture is non-intuitive for statistical analysis
- No native error quantification (manual implementation required)
- Clustering tools not integrated with ML-ready exports
- Limited volumetric interactivity (no programmatic projection extraction)

CRISP Advantage: Full Python API under CC BY-NC-SA 4.0 license with interactive volumetric projections and SOAP-FPS subsampling for direct ML workflows.

MDAnalysis vs. CRISP

MDAnalysis excels at trajectory analysis and is widely used, but:

- Dynamic RDF analysis requires manual configuration
- Error analysis requires external statsmodels
- No volumetric analysis (custom code needed)
- API inconsistencies (AtomGroup vs. NumPy returns)
- MSD needs custom code for windowed ensemble averaging and directional components
- H-bond doesn't have the Network (graph theory) analysis

CRISP Advantage: Unified API across 11 modules with windowed ensemble MSD, directional diffusion analysis, and dynamic RDF/H-bond network, as well as similar detailed analysis generation without custom scripting.

ASE Native Modules vs. CRISP

ASE excels at simulation setup and structure manipulation, but trajectory analysis is limited:

- Minimal trajectory tools (basic I/O only)
- RDF implementation bugs (heteroatom pair normalisation issues)
- No statistical framework (no error propagation or convergence checking)
- Absent clustering tools and interactive visualisation

CRISP Advantage: Full ASE interoperability with species-specific indexing, SOAP-FPS subsampling, and direct trajec-

tory export without format conversion for use in MACE/SchNet/NequIP.

Furthermore, beyond these feature-level distinctions, CRISP delivers orders of magnitude improvements in computational performance for equivalent analysis tasks. Table 2 provides a quantitative comparison of execution times for a representative Oxygen-Oxygen Radial Distribution Function (RDF) analysis on a 1,248-atom system over 440 frames. As explored in deeper detail in Section 3.5.1 (see Figure 21), CRISP achieves a base algorithmic speedup of 40.5 times on a single core and scales to over $1,300$ times when parallelised across 64 cores.

TABLE 2 Quantitative performance scaling of the CRISP `prdf.py` module versus native ASE for an O-O Radial Distribution Function (1,248 atoms, 440 frames). Evaluated on an AMD EPYC 9554 HPC workstation.

Framework	CPU Cores	Wall-Clock Time (s)	CRISP Speedup vs. ASE
Native ASE	1	12045.53	1.0×
CRISP	1	297.46	40.5×
CRISP	2	150.79	79.9×
CRISP	4	78.06	154.3×
CRISP	8	41.81	288.1×
CRISP	16	20.99	573.9×
CRISP	32	11.93	1009.6×
CRISP	64	9.22	1307.0×

These capabilities address ecosystem gaps in uncertainty quantification, ML-ready subsampling, clustering-guided diversity selection, and atomic volumetric analysis. Also, the open-source, modular nature of CRISP allows it to be flexible. All claims are verifiable via repository examples, documentation, and tests.

2 | SOFTWARE DESCRIPTION

This section provides detailed instructions for installation and links to essential tutorials.

2.1 | Installation and Tutorials

The section `https://crisp.readthedocs.io/en/latest/` includes detailed steps for installation and the required software packages.

The CRISP GitHub repository hosts a series of interactive tutorials designed to guide new users through the software's capabilities. These tutorials are provided as Jupyter notebooks and cover a range of topics relevant to molecular simulations and data analysis.

The tutorials to explain the modules of CRISP are available at **example** folder at GitHub:
`https://github.com/Indranil17/CRISP`.
At `example/Introductory_Tutorial`, tutorial in a Jupyter notebook format is available, providing an overview of the basic functionalities offered by CRISP. Each tutorial introduces a specific feature of the package, guiding one through

typical use cases.

The specific tutorials to explain the case study modules of CRISP are available at: `example/Specific_Tutorial`. The details of specific tutorials available in a Jupyter notebook format are as follows:

- **Subsampling:** A demonstration of the subsampling to select diverse subsets of a dataset. The notebook details the code for the rMD17 datasets case study in Section 3.2.
- **Error Analysis:** Tools and methods for quantifying uncertainties in molecular simulation data. The notebook details the code for the MFI Zeolite Chemical Shift Analysis case study in Section 3.1
- **Clustering:** Clustering method-DBSCAN to identify changes in atomic clusters during a simulation. The notebook details the code for the Platinum Migration in Zeolites case study in Section 3.3
- **H-Bond Analysis and Visualization :** Identification and visualisation of hydrogen bonding networks. The notebook details the code for the Bulk water case study in Section 3.4.
- **RDF (Radial Distribution Function):** Calculation and interpretation of partial radial distribution functions. Same as before, the study is on the Bulk water case study in Section 3.4.

Each tutorial is self-contained and includes example datasets, expected outcomes, and tips for further exploration.

2.2 | Architecture

Figure 2 summarises the detailed source code statistics and distribution for CRISP.

Language	files	blank	comment	code
CSV	12	0	0	109820
HTML	17	8558	0	70810
Python	47	2159	2669	7370
Jupyter Notebook	3	0	6384	691
reStructuredText	14	648	900	597
Markdown	7	82	1	245
YAML	8	29	27	191
TOML	1	13	1	61
DOS Batch	1	8	1	27
make	1	4	6	10
Bourne Again Shell	1	2	2	3
SUM:	112	11503	9991	189825

FIGURE 2 The overview of the CRISP source code characteristics - counted blank lines, comment lines, and physical lines. Scientific functionality is implemented entirely in Python, with Jupyter Notebooks for reproducible tutorials. Other auxiliary languages and file types are for package handling, documentation, and deployment infrastructure, sometimes inherited from web hosting templates.

The full HTML coverage report and test outputs are included in the Figure below.

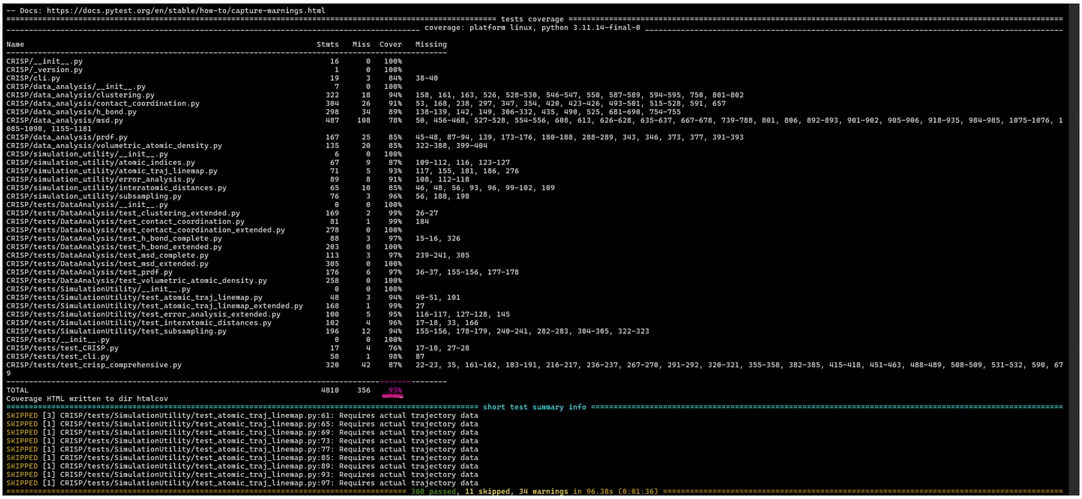

```
-- Docs: https://docs.pytest.org/en/stable/how-to/capture-warnings.html
============================================================ tests coverage ============================================================
____________________________________________ coverage: platform linux, python 3.11.14-final-0 ____________________________________________

Name                                                              Stmts   Miss  Cover   Missing
-----------------------------------------------------------------------------------------------
CRISP/__init__.py                                                    16      0   100%
CRISP/_version.py                                                     1      0   100%
CRISP/cli.py                                                         19      3    84%   38-40
CRISP/data_analysis/__init__.py                                       7      0   100%
CRISP/data_analysis/clustering.py                                   322     18    94%   158, 161, 163, 526, 528-530, 546-547, 550, 587-589, 594-595, 750, 801-802
CRISP/data_analysis/contact_coordination.py                         304     26    91%   53, 168, 238, 297, 347, 354, 420, 423-426, 493-501, 515-528, 591, 657
CRISP/data_analysis/h_bond.py                                       298     34    89%   138-139, 142, 149, 306-332, 435, 490, 525, 681-690, 754-755
CRISP/data_analysis/msd.py                                          487    108    78%   50, 456-468, 527-528, 554-556, 608, 613, 626-628, 635-637, 667-678, 739-788, 801, 806, 892-893, 901-902, 905-906, 918-935, 984-985, 1075-1076, 1
085-1098, 1155-1181
CRISP/data_analysis/prdf.py                                         167     25    85%   45-48, 87-94, 139, 173-176, 180-188, 288-289, 343, 346, 373, 377, 391-393
CRISP/data_analysis/volumetric_atomic_density.py                    135     20    85%   322-388, 399-404
CRISP/simulation_utility/__init__.py                                  6      0   100%
CRISP/simulation_utility/atomic_indices.py                           67      9    87%   109-112, 116, 123-127
CRISP/simulation_utility/atomic_traj_linemap.py                      71      5    93%   117, 155, 181, 186, 276
CRISP/simulation_utility/error_analysis.py                           89      8    91%   108, 112-118
CRISP/simulation_utility/interatomic_distances.py                    65     10    85%   46, 48, 56, 93, 96, 99-102, 109
CRISP/simulation_utility/subsampling.py                              76      3    96%   56, 188, 198
CRISP/tests/DataAnalysis/__init__.py                                  0      0   100%
CRISP/tests/DataAnalysis/test_clustering_extended.py                169      2    99%   26-27
CRISP/tests/DataAnalysis/test_contact_coordination.py                81      1    99%   184
CRISP/tests/DataAnalysis/test_contact_coordination_extended.py      278      0   100%
CRISP/tests/DataAnalysis/test_h_bond_complete.py                     88      3    97%   15-16, 326
CRISP/tests/DataAnalysis/test_h_bond_extended.py                    203      0   100%
CRISP/tests/DataAnalysis/test_msd_complete.py                       113      3    97%   239-241, 305
CRISP/tests/DataAnalysis/test_msd_extended.py                       305      0   100%
CRISP/tests/DataAnalysis/test_prdf.py                               176      6    97%   36-37, 155-156, 177-178
CRISP/tests/DataAnalysis/test_volumetric_atomic_density.py          258      0   100%
CRISP/tests/SimulationUtility/__init__.py                             0      0   100%
CRISP/tests/SimulationUtility/test_atomic_traj_linemap.py            48      3    94%   49-51, 101
CRISP/tests/SimulationUtility/test_atomic_traj_linemap_extended.py  168      1    99%   27
CRISP/tests/SimulationUtility/test_error_analysis_extended.py       100      5    95%   116-117, 127-128, 145
CRISP/tests/SimulationUtility/test_interatomic_distances.py         102      4    96%   17-18, 33, 166
CRISP/tests/SimulationUtility/test_subsampling.py                   196     12    94%   155-156, 178-179, 240-241, 282-283, 304-305, 322-323
CRISP/tests/__init__.py                                               0      0   100%
CRISP/tests/test_CRISP.py                                            17      4    76%   17-18, 27-28
CRISP/tests/test_cli.py                                              58      1    98%   87
CRISP/tests/test_crisp_comprehensive.py                             320     42    87%   22-23, 35, 161-162, 183-191, 216-217, 236-237, 267-270, 291-292, 320-321, 355-358, 382-385, 415-418, 451-463, 488-489, 508-509, 531-532, 590, 67
9
-----------------------------------------------------------------------------------------------
TOTAL                                                              4810    356    93%
Coverage HTML written to dir htmlcov
======================================================== short test summary info ========================================================
SKIPPED [3] CRISP/tests/SimulationUtility/test_atomic_traj_linemap.py:61: Requires actual trajectory data
SKIPPED [1] CRISP/tests/SimulationUtility/test_atomic_traj_linemap.py:65: Requires actual trajectory data
SKIPPED [1] CRISP/tests/SimulationUtility/test_atomic_traj_linemap.py:69: Requires actual trajectory data
SKIPPED [1] CRISP/tests/SimulationUtility/test_atomic_traj_linemap.py:73: Requires actual trajectory data
SKIPPED [1] CRISP/tests/SimulationUtility/test_atomic_traj_linemap.py:77: Requires actual trajectory data
SKIPPED [1] CRISP/tests/SimulationUtility/test_atomic_traj_linemap.py:85: Requires actual trajectory data
SKIPPED [1] CRISP/tests/SimulationUtility/test_atomic_traj_linemap.py:89: Requires actual trajectory data
SKIPPED [1] CRISP/tests/SimulationUtility/test_atomic_traj_linemap.py:93: Requires actual trajectory data
SKIPPED [1] CRISP/tests/SimulationUtility/test_atomic_traj_linemap.py:97: Requires actual trajectory data
=============================== 360 passed, 11 skipped, 34 warnings in 96.38s (0:01:36) ===============================
```

FIGURE 3 Unit test coverage summary for the `CRISP` codebase computed with `pytest`. Total line coverage is approximately 93% (360 passing tests). Per module data is shown: simulation utility modules (`atomic_indices`, `interatomic_distances`, `subsampling` and `error_analysis`) now range between 85% and 96%; data analysis modules (`msd`, `h_bond`, `clustering`, `volumetric_atomic_density` and `contact_coordination`) range between 78% and 94%; core infrastructure and CLI achieve 84% to 100% coverage.

Below, we provide the Structural Overview for the package. We provide two diagrams for two subpackages. The idea is to describe with a graphical notation classes, keywords, inputs and the relationships between them.

Simulation Utility

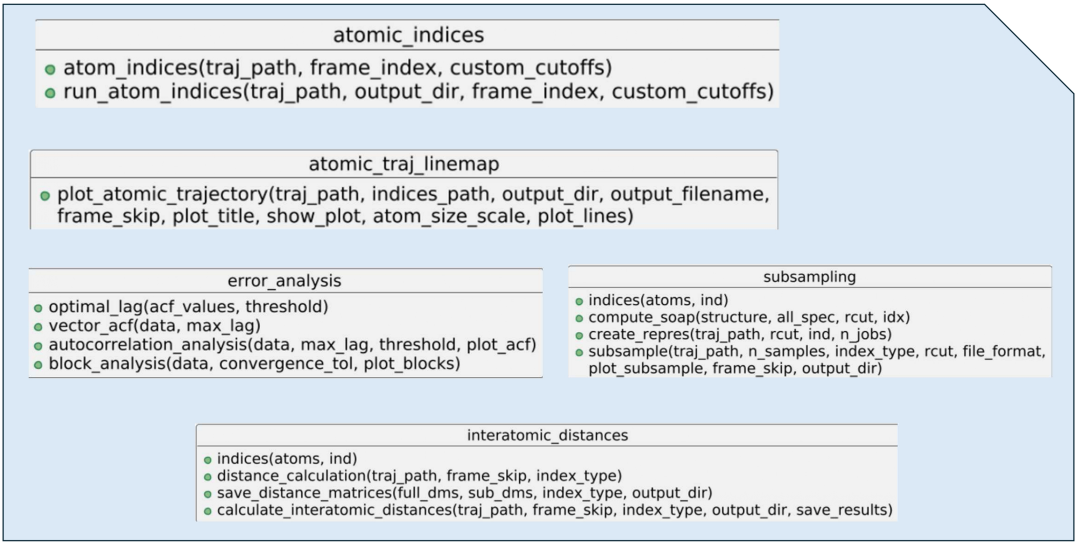

FIGURE 4 Structural Overview diagram illustrating the functions within the CRISP **Simulation Utility** sub-package. This sub-package handles trajectory preprocessing through modules for error validation, subsampling, and more.

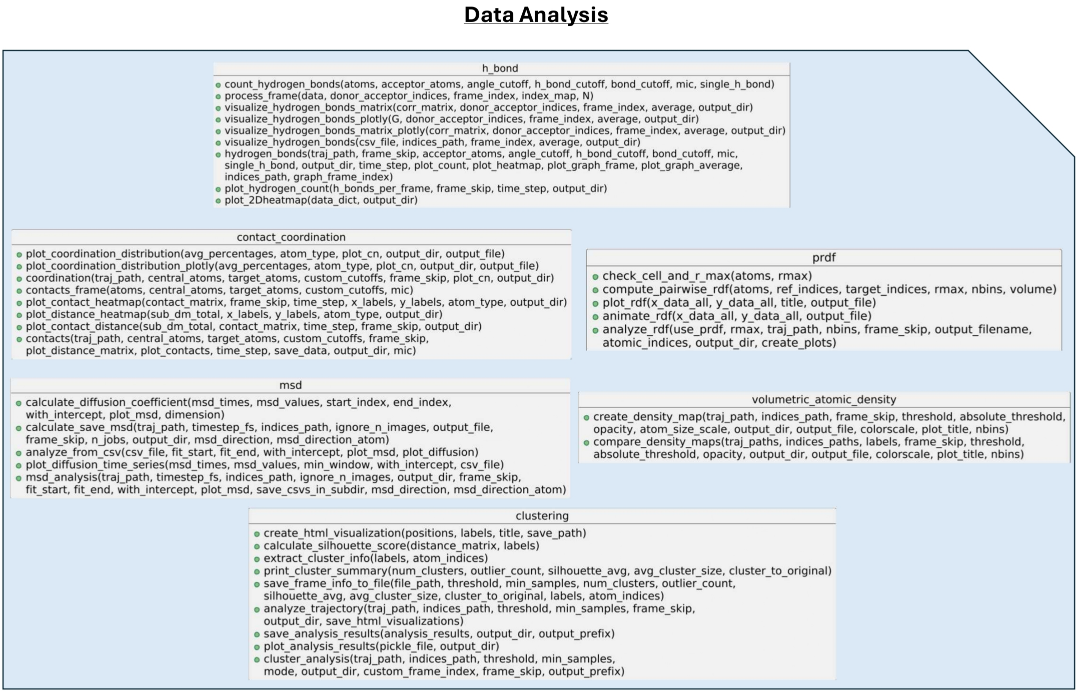

FIGURE 5 Structural Overview diagram illustrating the functions within the CRISP **Data Analysis** sub-package. This sub-package extracts chemical insights using modules for clustering, hydrogen bond analysis, spatial distribution, and more.

2.3 | Functionalities Implementation Overview

This section summarises the capabilities provided by the **simulation_utility** and **data_analysis** subpackages, which implement minimum-image distance handling [3], SOAP/FPS sampling [4], statistical error analysis [5], and a suite of trajectory analysis and visualisation tools leveraging standard methods and interactive plotting [6].

2.3.1 | Simulation Utility

The functionalities of the modules belonging to the **simulation_utility** subpackage are as follows,

1. **atomic_indices.py:** Provides tools for extracting atomic indices based on chemical symbols or other custom criteria, saving the results for use in other analyses. The module works on a single frame of a trajectory at a time, and also computes the minimum-image-convention[3] distance matrix between all atoms and atomic pairs within a custom cut-off if required.
2. **interatomic_distances.py:** Calculates and stores distance matrices for trajectory (for all or only selected atoms), with capabilities to apply periodic boundary conditions. In essence, it reads the requested number of frames, skipping the user-specified number of frames, and computes the full $(N \times N)$ distance matrix for each frame using the minimum-image convention, using SI Equation 1 if required. It also allows the possibility of generating a distance matrix for a subset of atoms.

3. **subsampling.py:** Implements Furthest Point Sampling (FPS) with Smooth Overlap of Atomic Positions (SOAP) descriptor[4] to select representative structures from a simulation trajectory or a structural database. Furthest Point Sampling (FPS) is a greedy algorithm used to extract a diverse subset of structures from a larger dataset.
4. **error_analysis.py:** Provides statistical error analysis for molecular dynamics simulations by implementing auto-correlation function in the statsmodels package[5] and block averaging methods to quantify uncertainty in time-correlated simulation data properly.
5. **atomic_traj_linemap.py:** Generates interactive 3D visualisations (using Plotly package[6]) of atomic trajectories from molecular simulations, displaying the movement paths of selected atoms.

2.3.2 | Data Analysis

Next, in the **data_analysis** sub-package, the functionalities of the modules are as follows,

1. **clustering.py:** Employs the DBSCAN algorithm [7] to cluster atomic structures and computes summary statistics such as the silhouette score [8] and cluster sizes for both individual frames and whole trajectories. Interactive 3D visualisations of clusters are generated.
2. **contact_correlation.py:** Analyses coordination numbers and contact (boolean) within a predefined cut-off between specified "central" atom(s) and selected "target" atoms. As an output, using van der Waals radii and custom cut-off values, the module computes adjacency/contact as well as distance matrices. For each frame (k), the distance $D_{ij}^{(k)}$ is calculated (using SI Equation 1) between a set of “central” atoms (i) and “target” atoms (j). Then, based on either the default van der Waals radii of a coordination contact ($0.6\times(R_i^{\mathrm{VdW}} + R_j^{\mathrm{VdW}})$) or custom cutoff supplied for pair (i, j), a contact is defined $M_{ij}^{(k)}$ to be 1 else no contact 0.
3. **h_bond.py:** Focuses on hydrogen bond analysis by calculating correlation matrices, constructing network graphs, and generating both static and interactive visualisations of hydrogen bond dynamics. The code identifies hydrogen bonds by examining Donor–Hydrogen–Acceptor (D–H–A) against user-defined geometric criteria or default values, in agreement with the IUPAC definition[9].
4. **msd.py:** Calculates the Mean Square Displacement (MSD) and diffusion coefficients from trajectory data. The module allows two widely adopted approaches for MSD calculation (single origin and windowed/multiple time origin, described in detail in the SI section 2.4). Then it uses Einstein’s relation [10] to calculate the diffusion coefficient and produces a time-evolution plot of the diffusion coefficient.
5. **prdf.py:** Performs (partial) radial distribution function analysis[11]. The module includes static plotting and animation capabilities to visualise the evolution of local structures in the system. For each frame k, extract the minimum-image distances ($D_{ij}^{(k)}$) between reference atoms $i \in A$ and target atoms $j \in B$. Bin all distances into a histogram $\mathrm{hist}(r)$ over $[0, r_{\max}]$ with bin width Δr.
6. **volumetric_atomic_density.py:** Builds an interactive 3D density map of selected atoms by binning their wrapped positions $\{\mathbf{r}_i^{(k)}\}$ over all frames into a regular grid[12]. First, it collects positions for atom indices I in each frame $k = 1 \ldots M$. Then, it computes a 3D histogram for each direction, binning atoms along each direction axis into a specific bin(s) H_{abc}.

2.4 | Functionalities Implementation Details

This section provides implementation-level notes for each module in the **Simulation_Utility** and **data_analysis** sub-packages.

In the **Simulation_Utility** sub-package, the details of the functionalities of the modules are as follows,

1. **atomic_indices.py:** This module calculates the distance between atoms and generates atom-specific data.

$$d_{ij} = \min_{\mathbf{n}\in\mathbb{Z}^3} \left\| \mathbf{r}_i - \mathbf{r}_j + \mathbf{n}\cdot\mathbf{L} \right\| \tag{1}$$

where d_{ij} is distance between atom i and atom j, and $\mathbf{L}$ is the periodic cell matrix. Then builds two outputs:
1. A dictionary $\{A \mapsto S_A\}$ mapping each chemical symbol A to the list of atom indices S_A.
2. For each user-supplied cutoff r_c^{AB} for a symbol pair (A, B), the set of all index-distance tuples,

$$\{(i, j, d_{ij}) \mid i \in S_A,\ j \in S_B,\ d_{ij} < r_c^{AB}\} \tag{2}$$

is generated and written to CSV for downstream filtering or classification. This simple but flexible scheme lets you extract “all oxygens,” “all hydrogens,” or any custom bonded pairs (e.g. O–Al, H–O) in one pass.

2. **interatomic_distances.py:** This module is about calculating and managing interatomic distance matrices.

$$D_{\mathrm{sub}}^{(k)} = D^{(k)}[\mathrm{idx}, , \mathrm{idx}] \tag{3}$$

Here, $D^{(k)}$ represents the full distance matrix for all atoms at snapshot k, and idx is an array or list containing the indices of the atoms of interest (e.g., indices corresponding to "all O atoms" or loaded from an .npy index file). While the $D_{\mathrm{sub}}^{(k)}$ is for a sub-matrix of interatomic distances for a specific group of atoms. This operation effectively extracts the pairwise distances only between the atoms specified by ‘idx’. It returns the distance matrix (either the full ones or selected subsets) using Python lists in pickle (.pkl) files, which can be used for downstream analyses (RDF, CN, etc.). By having at disposal both full and indexed distances, one obtains both a total view of all interatomic distances and a view focused on atoms of interest.

3. **subsampling.py:** Subsampling employs the Farthest point sampling algorithm (FPS)[13], which starts from an initial randomly selected point x_0, and each subsequent point x_i is chosen such that it maximises its minimum distance to the already selected points. Formally, given a distance metric $d(x, y)$, the i-th point is chosen as

$$x_i = \arg\max_{x\in X} \min_{x_j\in S} d(x, x_j) \tag{4}$$

where X is the full dataset and S is the set of previously selected points. When using SOAP descriptors to define the distance metric, FPS efficiently identifies the most structurally diverse configurations. This method reduces redundancy in the dataset while preserving the chemical variability. In CRISP’s implementation, the SOAP descriptors of all or selected atoms are averaged to obtain a single descriptor representing the entire structure.[4, 14]

4. **error_analysis.py:** Autocorrelation analysis begins by computing the autocorrelation function $C(\tau)$ of an observable $A(t)$ as a function of (time) lag τ.[15] Formally, one defines

$$C(\tau) = \frac{\left\langle \left(A(t) - \bar{A}\right)\left(A(t+\tau) - \bar{A}\right)\right\rangle}{\left\langle \left(A(t) - \bar{A}\right)^2 \right\rangle}, \tag{5}$$

where $\bar{A} = \langle A(t) \rangle$ is the time-average of A. The integrated autocorrelation time τ_{int} is then obtained by summing $C(\tau)$

$$\tau_{\text{int}} = \Delta t \sum_{\tau=0}^{\tau_{\text{cutoff}}} C(\tau), \quad C(\tau_{\text{cutoff}}) < 0.05, \tag{6}$$

with Δt the simulation time step. This threshold 0.05 was chosen to exclude correlations dominated by noise.[16] We regard samples separated by at least τ_{int} as effectively uncorrelated, and we deem a trajectory of total length T to be converged when

$$T \geq 20\,\tau_{\text{int}}. \tag{7}$$

The factor of 20 is chosen based on the rule of thumb that the total simulation length should be at least 20 times the integrated autocorrelation time to ensure reliable statistical sampling, which is generally considered more than adequate according to Sokal[16]. The standard error of the mean is then defined as

$$\sigma_{\bar{A}} = \sqrt{\frac{2\,\sigma_A^2\,\tau_{\text{int}}}{N}} = \sqrt{\frac{\sigma_A^2}{N_{\text{eff}}}},$$

where N_{eff} is the number of uncorrelated samples. Block averaging provides an alternative estimate of the standard error by partitioning the trajectory of length T into M contiguous blocks, each of duration $B = T/M$. One computes the block means $\{\mu_k\}$ and then the block-averaged variance

$$\sigma_{\text{block}}^2 = \frac{1}{M(M-1)} \sum_{k=1}^{M} (\mu_k - \bar{\mu})^2, \tag{8}$$

where $\bar{\mu} = \frac{1}{M} \sum_k \mu_k$. The block size B is increased until σ_{block} converges, defined as a change smaller than 10^{-4} between successive block sizes.

5. **atomic_traj_linemap.py:** It provides functionality for visualising atomic trajectories, plotting the paths of selected atoms. Given a trajectory and an index list $\{i\}$, it reads frames $\{\mathbf{r}^{(k)}\}$, adopts the size of markers given by Van der Waals radii[17] $R_{\text{VDW}}(A)$ via $\text{size}_i \propto R_{\text{VDW}}(\text{symbol}_i) \times \text{scale}$, and colours them by element. For each atom i, its path $\{\mathbf{r}_i^{(k)}\}$ is plotted either as discrete points or as line segments connecting subsequent frames $\mathbf{r}_i^{(k)} \to \mathbf{r}_i^{(k+1)}$. The result is saved as a standalone HTML [6] file for interactive exploration.

In the **data_analysis** sub-package, the details of the functionalities of the modules are as follows,

1. **clustering.py:** This module applies the DBSCAN algorithm to identify and analyse atomic clusters within a simulation. The DBSCAN algorithm[18] is applied on each frame's precomputed distance matrix D_{ij} (mic-corrected). For each point i, define the set of neighbors within threshold ε as,

$$N_\varepsilon(i) = \{j : D_{ij} \leq \varepsilon\} \tag{9}$$

A point i is a core point if the $N_\varepsilon(i)$, number of neighbours (including itself) is at least m, i.e.,

$$|N_\varepsilon(i)| \geq m \tag{10}$$

where ε is the `eps` threshold distance between points/atoms and m is `min_samples`, the minimum number of atoms that makes a cluster. Two points i and j are density-connected if there exists a sequence of points such that each consecutive pair is within distance ε of each other (not further than one neighbour relation away). Thus, clusters are then maximal sets of density-connected points. Points assigned to no cluster are labelled as outliers. For each frame (or the whole trajectory), the module returns: cluster labels $\{l_i\}$ and outlier count, number of clusters and their sizes $\{|C_k|\}$, silhouette score (S), interactive visualisations of atoms colored by cluster and a collage plot of the variables changing ($\{|C_k|\}$ and S) during frames of the trajectory.

2. **contact_correlation.py:** We compute the instantaneous contacts and coordination numbers as,

$$\mathrm{CN}_i^{(k)} = \sum_{j \in \mathcal{T}} M_{ij}^{(k)}, \quad i \in C \tag{11}$$

where, $\mathrm{CN}_i^{(k)}$ is the coordination number of central atom i at frame k. C and $\mathcal{T}$ is the set of central and target atoms respectively. Finally, $M_{ij}^{(k)}$ is the contact matrix element, equal to 1 if atoms i and j are in contact at frame k (i.e., their distance is less than the cutoff), and 0 otherwise.
The module then generates the following: time-series and pie-chart distributions of CNs, contact-time heatmaps $T_{ij} = \sum_k M_{ij}^{(k)} \Delta t$, distance heatmaps $\langle D_{ij} \rangle$, and interactive 3D plots of contacts/coordination for further inspection.

3. **h_bond.py:** It identifies hydrogen bonds in molecular dynamics trajectories by evaluating geometric criteria for each possible hydrogen–acceptor pair in every frame. Specifically, for each frame k, the hydrogen bond indicator function $I_{H,A}^{(k)}$ is defined as

$$I_{H,A}^{(k)} = \begin{cases} 1, & d_{H,A}^{(k)} < d_c \ \wedge \ \theta_{DHA}^{(k)} > \theta_c, \\ 0, & \text{otherwise,} \end{cases} \tag{12}$$

where $d_{H,A}$ is the threshold H-acceptor distance and θ_{DHA} the threshold D–H–A angle (minimum-image convention via Equation 1), also it uses the default as per the IUPAC definition[9]. For each frame k, the module computes the total number of hydrogen bonds per frame (k) $N_{\mathrm{Hbond}}^{(k)} = \sum_{H,A} I_{H,A}^{(k)}$ and stores the total count for each atomic pair that is hydrogen-bonded as $C_{ij} = \sum_k I_{i,j}^{(k)}$.
Lastly, it produces the following outputs: per-frame bond counts $N_{\mathrm{Hbond}}(t)$ saved in a CSV file, 2D histograms of θ versus d (`plot_2Dheatmap`), donor-acceptor correlation matrix and weighted connected graph (`visualize_hydrogen_bonds`), time-series plots (`plot_hydrogen_count`) and interactive network/heatmap outputs.

4. **msd.py:**
This module computes the mean squared displacement (MSD) of atoms in a molecular dynamics trajectory. For a

set of N atoms, the MSD is defined as

$$\begin{aligned} \mathrm{MSD}(t_0, \tau) &= \frac{1}{N}\sum_{i=1}^{N} |\mathbf{r}_i(t_0+\tau) - \mathbf{r}_i(t_0)|^2 \\ \mathrm{MSD}_\alpha(t_0, \tau) &= \frac{1}{N}\sum_{i=1}^{N} \left[r_{i,\alpha}(t_0+\tau) - r_{i,\alpha}(t_0)\right]^2, \quad \alpha \in \{x, y, z\} \end{aligned} \tag{13}$$

where $\mathbf{r}_i(t_0)$ is the position of atom i at time t_0, and τ is the lag time.

Two widely adopted MSD calculation methods[19] are available:

a. **Single Origin Approach:** The reference time t_0 is fixed (typically $t_0 = 0$ or any $0 \le t_0 < T$, where T is the total number of frames). The MSD is computed as

$$\mathrm{MSD}(\tau) = \frac{1}{N}\sum_{i=1}^{N} |\mathbf{r}_i(t_0+\tau) - \mathbf{r}_i(t_0)|^2$$

b. **Windowed (Multiple Time Origin) Approach:** The MSD is averaged over all possible time origins t_0 for each lag time τ:

$$\mathrm{MSD}(\tau) = \frac{1}{M}\sum_{t_0=0}^{M} \left[\frac{1}{N}\sum_{i=1}^{N} |\mathbf{r}_i(t_0+\tau) - \mathbf{r}_i(t_0)|^2\right]$$

where M is the number of valid time origins for lag τ.

The windowed approach provides improved statistical reliability by averaging over all possible starting points in the trajectory.

To obtain the diffusion coefficient, the plot of MSD vs. time is linearly fitted and the diffusion coefficient is extracted from the slope of the linear fit, $D = \frac{1}{2d}\frac{d\,(\mathrm{MSD})}{dt}$, where d is the system dimension. This module supports per-species or custom index subsets, CSV export, and time-series plotting.

5. **prdf.py:** The RDF ($g_{AB}(r)$) is obtained via

$$g_{AB}(r) = \frac{\mathrm{hist}(r)}{4\pi\, r^2\, \Delta r}\, \frac{1}{N_B\ (N_A/V)} \tag{14}$$

where N_A, N_B are the counts of reference and target atoms respectively, V is the cell volume. Δr is the histogram bin width. The $\mathrm{hist}(r)$ is the number of atom pairs with separation in the interval $[r, (r + \Delta r)]$.

The RDF is calculated for all (selected) frames (k) in parallel, averaged to obtain $g(r)$ and saved as $\{r_k, g_k\}$ in a pickle file. Then, as part of the plotting, a static plot of $\langle g(r)\rangle$ with a peak marker is saved. Also, it saves an animated GIF/HTML showing frame-by-frame $g(r)$ evolution.

6. **volumetric_atomic_density.py:** This module computes the spatial distribution of selected atoms in a molecular dynamics trajectory by constructing a 3D histogram over the unit cell. For a set of M frames and a set I of selected atom indices, the density in each grid box (a, b, c) is defined as

$$H_{abc} = \sum_{k=1}^{M}\sum_{i\in I} \mathbb{1}\left(x_i^{(k)} \in [x_a, x_{a+1}),\ y_i^{(k)} \in [y_b, y_{b+1}),\ z_i^{(k)} \in [z_c, z_{c+1})\right), \tag{15}$$

where $x_i^{(k)}, y_i^{(k)}, z_i^{(k)}$ are the coordinates of atom i in frame k, and $\mathbb{1}(\cdot)$ is the indicator function (i.e., $\mathbb{1}(A) = 1$ if A is true, 0 otherwise). The grid spans the simulation cell, with $[x_a, x_{a+1})$ denoting the a-th bin along x, and similarly for y and z.

Optionally, the histogram can be normalised as $\tilde{H} = H/H_{\text{max}}$ to yield a relative density. The parameter `absolute_threshold` (ϵ) determines whether isosurfaces are rendered at $\tilde{H} \geq \epsilon$ (relative) or $H \geq \epsilon$ (absolute). The code visualises the density as a 3D isosurface (using a Plotly `Volume` trace), overlays the static reference structure (with marker sizes set by van der Waals radii), and exports the result as a standalone HTML file for interactive exploration.

2.5 | Workflow

In the example workflow, we first used `simulation_utility.error_analysis` to quantify the statistical uncertainty in the total energy employing autocorrelation and block-averaging error estimators. Next, we used `simulation_utility.run_atom_indices` routine to identify all indices by computing pairwise distances (O–Al, O–Si, H–O), saving index arrays for subsequent analyses that would require a classification among the framework and water atoms (oxygen, hydrogen atoms mapped as per their belonging). Also, in order to reduce the data redundancy, the trajectory was subsampled via farthest-point sampling on SOAP descriptors `simulation_utility.subsampling`, producing a small set of maximally diverse frames. Furthermore, as part of the data analysis, we computed both total RDFs and partial RDFs (O–O, O–H, and H–H) using `data_analysis.prdf` to extract coordination peaks and hydration structures around selected framework and water species. Diffusion coefficients were then obtained with `data_analysis.calculate_save_msd` module deployed on water oxygens, followed by linear fitting of the MSD curve. To obtain time-averaged CN distributions and a pie chart of types of CN, we evaluated water-oxygen coordination environments (water–water) using `data_analysis.coordination` routine. Finally, the `data_analysis.h_bond` module identified and counted hydrogen bonds (following the angle and distance criteria), assuming oxygen atoms as acceptors, producing per-frame statistics saved in CSVs and summary plots such as a time series line plot of hydrogen bonds, a 2D histogram, and a connected graph visualising the donor-acceptor atoms involved in the hydrogen bond.

The workflow is accessible in CRISP's GitHub repository as an interactive Jupyter notebook at `example/Workflow/collage_calculations.ipynb`.

3 | ILLUSTRATIVE EXAMPLES

3.1 | Case Study: MFI Zeolite, Error Analysis

The case study used to evaluate the prediction error was taken from our previous work.[20] It consists of a 1 ns molecular dynamics (MD) simulation with a time step of 0.5 fs. The ^{27}Al chemical shift was computed every 100 steps along the trajectory, resulting in 20,000 chemical shift predictions out of a total of 2 million MD steps. The simulation was carried out at 350 K using a Nosé–Hoover thermostat with a coupling time of 40 fs. This thermostat relaxation time is on the same order of magnitude as the observed autocorrelation time of approximately 50 fs for the chemical shift, consistent with the known influence of thermostat strength on dynamical correlation properties.

The system investigated is a zeolite MFI framework containing a single aluminium atom substituted at the T1 site and three water molecules per unit cell. This composition was selected due to its chemical relevance and previously observed sensitivity of the ^{27}Al shift to hydration and local environment.

3.2 | Case Study: rMD17 Dataset, Dataset Subsampling

The MACE models were trained using the following parameters:

```
r_max: 6.0
num_channels: 128
max_L: 0
```

These settings were chosen to match those of the small MACE foundational model.[21] Training was stopped after 20 epochs without improvement in validation loss.

Three models were trained on different datasets: (i) a randomly selected dataset of 10,000 structures provided by the rMD17 protocol (rMD17-10k),[22] (ii) a farthest point sampled (FPS) subset of 10,000 structures (FPS-10k), and (iii) an FPS subset of 1,000 structures (FPS-1k).

As shown in Table 3, models trained on FPS-subsampled datasets converge significantly faster while maintaining or even improving the accuracy of the predictions. The FPS models exhibit slightly better performance, which is expected to be even more pronounced in more diverse datasets. Since rMD17 consists of molecular dynamics-generated data, it contains substantial redundancy, and thus systematic subsampling provides a clear advantage.

Model	Energy [meV/atom]	Forces [meV/Å]	Epochs
rMD17-10k	2.8	32	144
FPS-10k	2.0	28	66
FPS-1k	2.2	34	137

TABLE 3 Test set mean absolute errors for energy and forces of MACE models trained on different rMD17-based datasets.

3.3 | Case Study: Platinum Migration In Zeolites

Our objectives in this case study were twofold: first, to verify the key findings reported by Heard et al. [23] regarding cluster behaviour and migration, and second, to demonstrate how CRISP can provide novel visualisations and quantitative insights complementary to the original study.

3.3.1 | Clustering

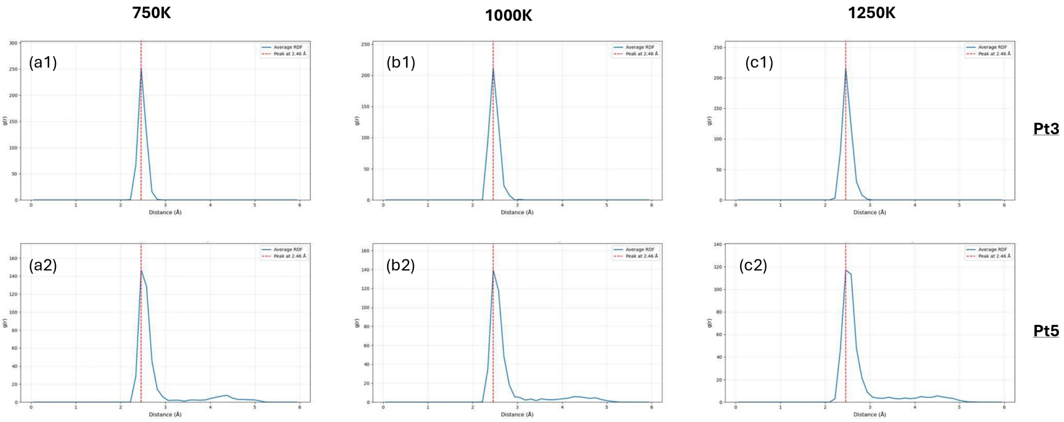

FIGURE 6 Pt–Pt pair radial distribution functions (RDFs) for Pt_3 (top row: a1/b1/c1) and Pt_5 (bottom row: a2/b2/c2) clusters in CHA zeolite at 750K, 1000K, and 1250K. Each panel shows the average RDF (blue) and the position of the first coordination peak (red dashed line, 2.46Å).

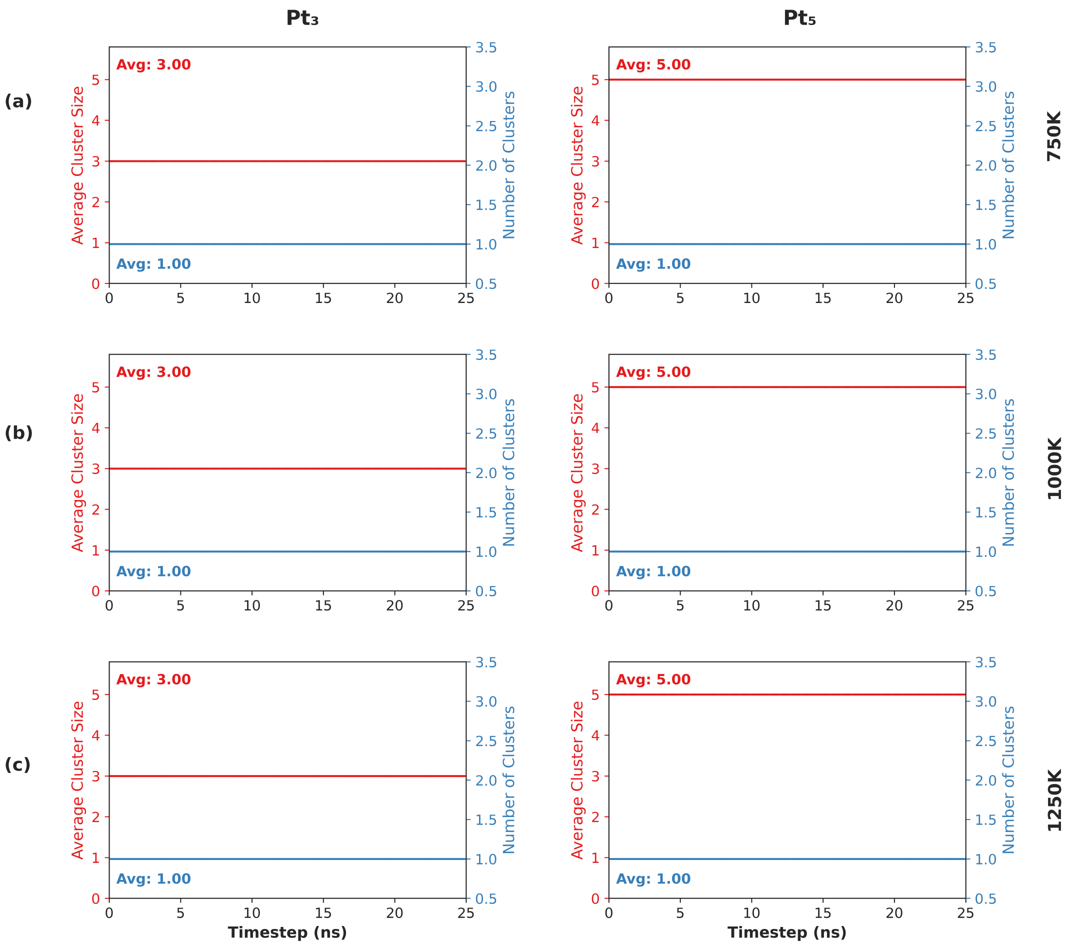

FIGURE 7 DBSCAN clustering analysis results for Pt_3 (left column) and Pt_5 (right column) clusters in CHA zeolite using a distance threshold ϵ = 3.0 Å and 'min_samples = 2'. Each plot shows the average cluster size (red, left y-axis) and the number of clusters (blue, right y-axis) as a function of simulation frame number at (a) 750 K, (b) 1000 K, and (c) 1250 K. Dashed lines indicate the average values over the trajectories, with corresponding numerical values displayed in matching colors.

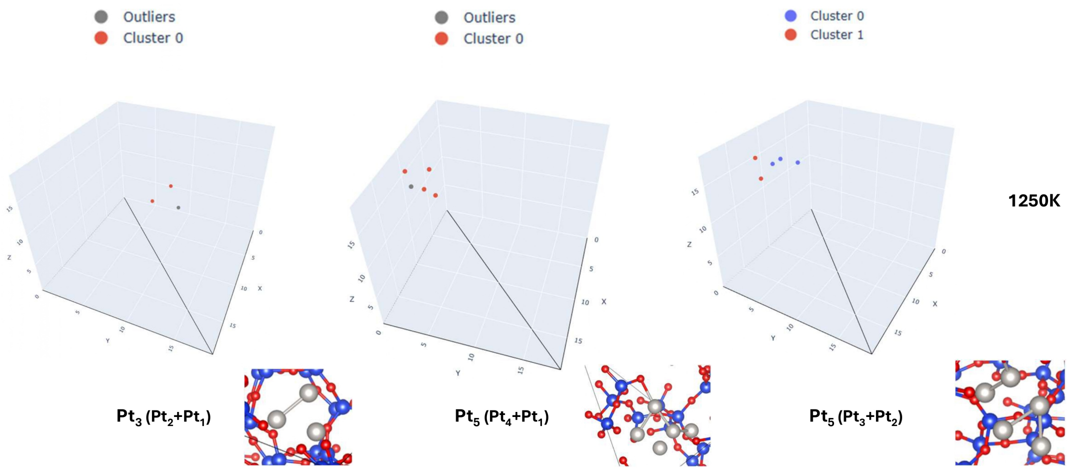

FIGURE 8 Representative snapshots illustrating cluster fragmentation dynamics at 1250 K, identified using DBSCAN (ϵ = 2.6 Å 'min_samples = 2').
(Left) Transient fragmentation of Pt_3 into Pt_2 (Cluster 0, red) and Pt_1 (Outlier, grey). (Middle) Fragmentation of Pt_5 into Pt_4 (Cluster 0, red) and Pt_1 (Outlier, grey). (Right) Fragmentation of Pt_5 into Pt_3 (Cluster 0, blue) and Pt_2 (Cluster 1, red). Upper panels show 3D scatter plots of Pt atom positions colored by cluster identity (outliers in grey); lower panels show corresponding atomic configurations within the CHA zeolite framework (Pt: silver, O: red, Si/Al: blue).

3.3.2 | Volumetric Atomic Density

To visualise the spatial distribution and confinement of the platinum species within the CHA zeolite framework over the course of the molecular dynamics simulations, we performed volumetric atomic density analysis. This technique maps the probability density of Pt atoms within the simulation cell, providing insights into their preferred locations and the extent of their movement.

Below, the detailed results are presented separately for Pt_1, Pt_3, and Pt_5 clusters:

Pt_1 (Single Atom)

The volumetric density maps (SI Figure 9) show that Pt_1 is strongly confined to the 6-membered rings (6MRs) of the CHA zeolite framework across all simulated temperatures (750 K, 1000 K, 1250 K). Negligible density is observed within the larger cage or channel system, indicating the Pt atom does not readily detach from the vicinity of the framework. As temperature increases, density clouds become slightly more diffuse and distributed among more 6MR sites, suggesting increased thermal motion and hopping between adjacent 6MRs. Even at 1250 K, Pt_1 remains localised within these 6MR regions, indicating no significant inter-cage migration. The 2D projections (SI Figure 10) corroborate this, showing high-probability spots in the XY, XZ, and YZ planes near 6MRs, with increased spread and number of occupied sites at higher temperatures.

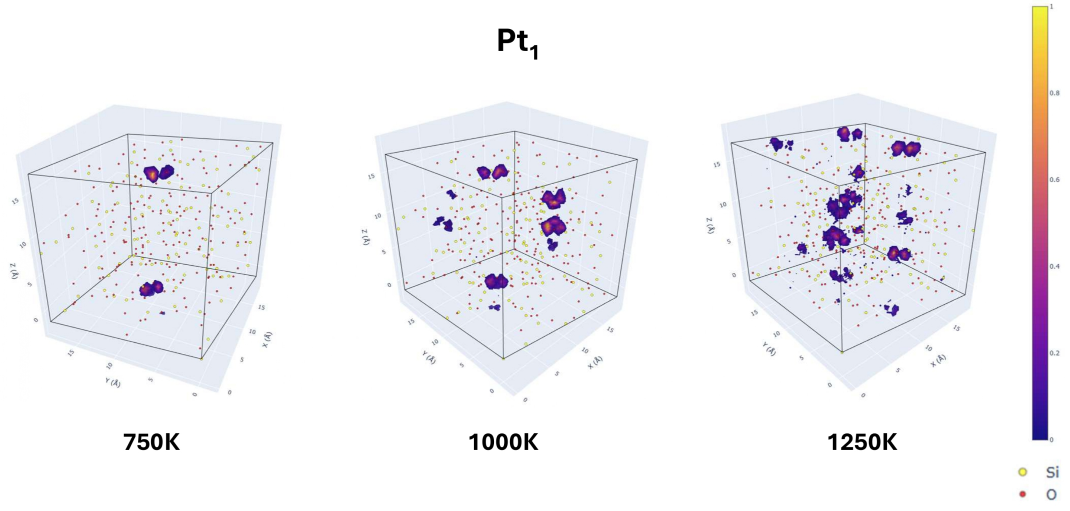

FIGURE 9 Volumetric density maps of Pt_1 (1 Pt atom in the unit cell) within the CHA zeolite framework at 750 K (left), 1000 K (middle), and 1250 K (right). Si and O atoms of the framework are shown as yellow and red spheres, respectively. The colour bar indicates probability density.

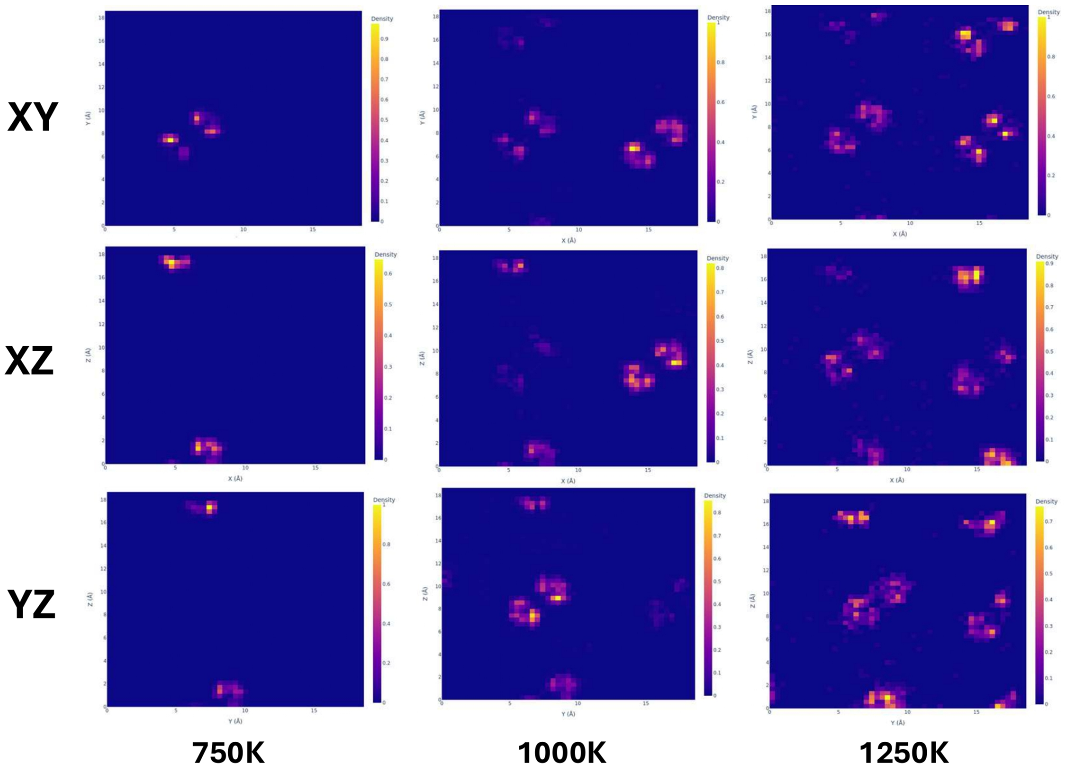

FIGURE 10 2D projections of Pt atomic density of Pt_1 within the CHA zeolite framework onto the XY, XZ, and YZ planes at 750 K, 1000 K, and 1250 K. Data obtained from molecular dynamics simulations. The colour bar indicates probability density, highlighting regions of higher Pt concentration.

Pt_3

The Pt_3 cluster exhibits significantly different behaviour compared to Pt_1. SI Figure 11 and SI Figure 12 indicate that at 750 K, the density map shows a single and relatively localised cluster occupying the larger 8-member ring (MR) cage. The density maps become noticeably more diffuse and elongated at elevated temperatures, suggesting increased thermal motion through the 8MR. These transient migration attempts are visible in both the 3D map and all 2D projections along the X and Z axes to the adjacent cages, with the clusters showing increased density near the centre of the CHA cage, especially at 1250 K. This temperature-dependent spatial evolution aligns with the liquid-like cluster dynamics observed in molecular simulations.

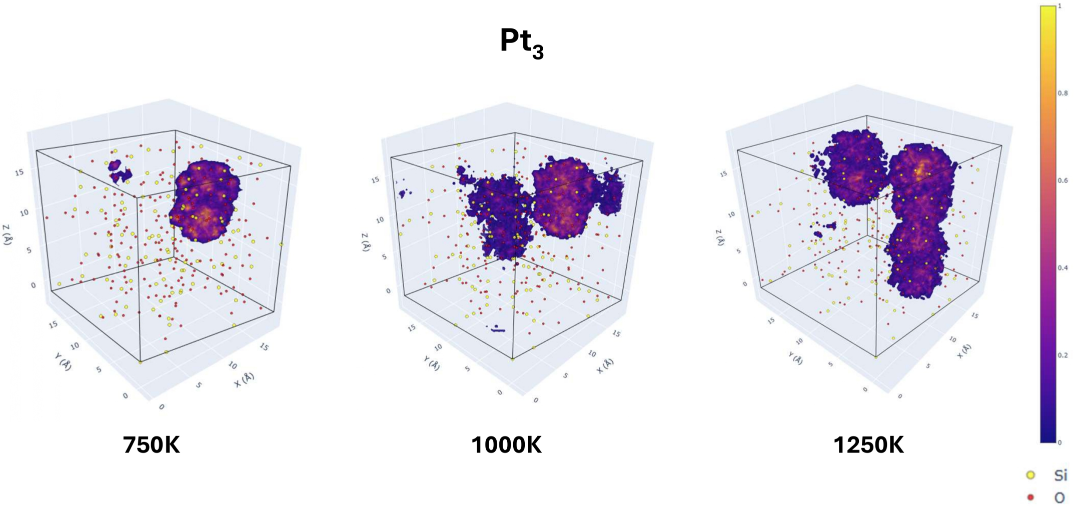

FIGURE 11 Volumetric density maps of Pt_3 (3 Pt atoms in the unit cell) within the CHA zeolite framework at 750 K (left), 1000 K (middle), and 1250 K (right). Si and O atoms of the framework are shown as yellow and red spheres, respectively. The colour bar indicates probability density.

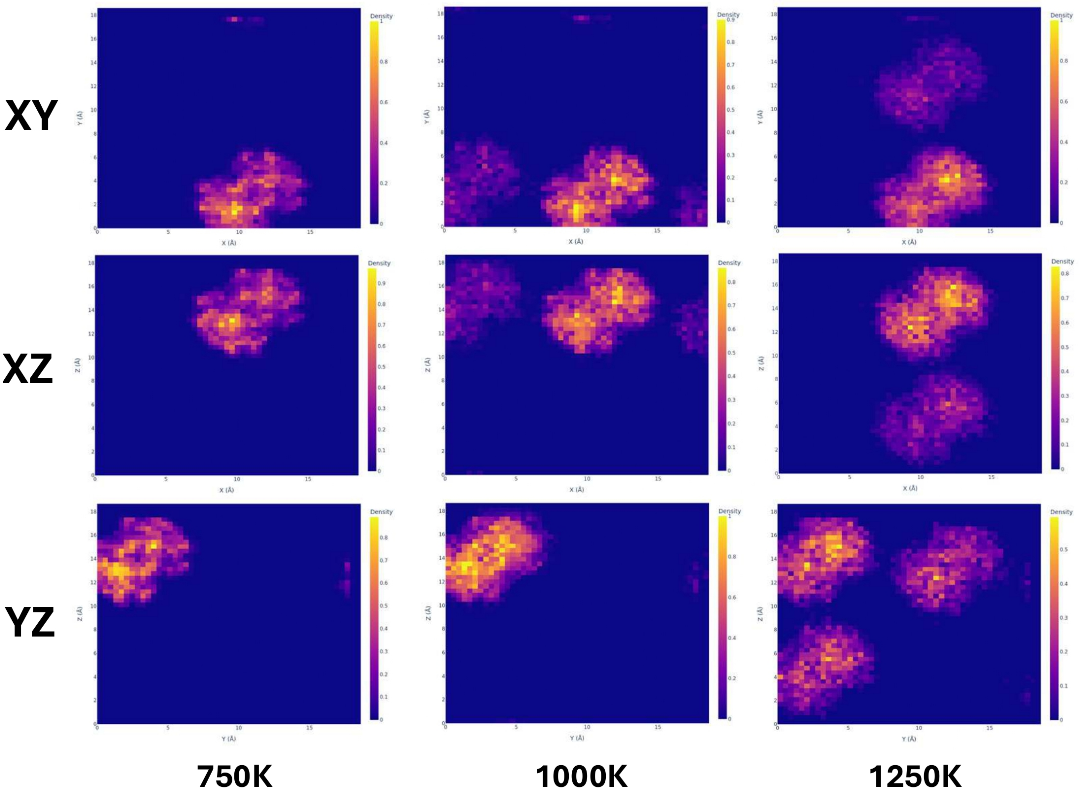

FIGURE 12 2D projections of Pt atomic density of Pt_3 within the CHA zeolite framework onto the XY, XZ, and YZ planes at 750 K, 1000 K, and 1250 K. Data obtained from molecular dynamics simulations. The colour bar indicates probability density, highlighting regions of higher Pt concentration.

Pt_5

The volumetric density maps (SI Figure 13) and 2D projections (SI Figure 14) demonstrate the strong confinement of Pt_5 clusters within CHA cages. At 750 K, the density distribution exhibits sharp localisation at cage centres, with no detectable density outside the confinement of CHA cage. The 2D projections confirm this strong confinement of Pt_5 clusters, even at elevated temperatures.

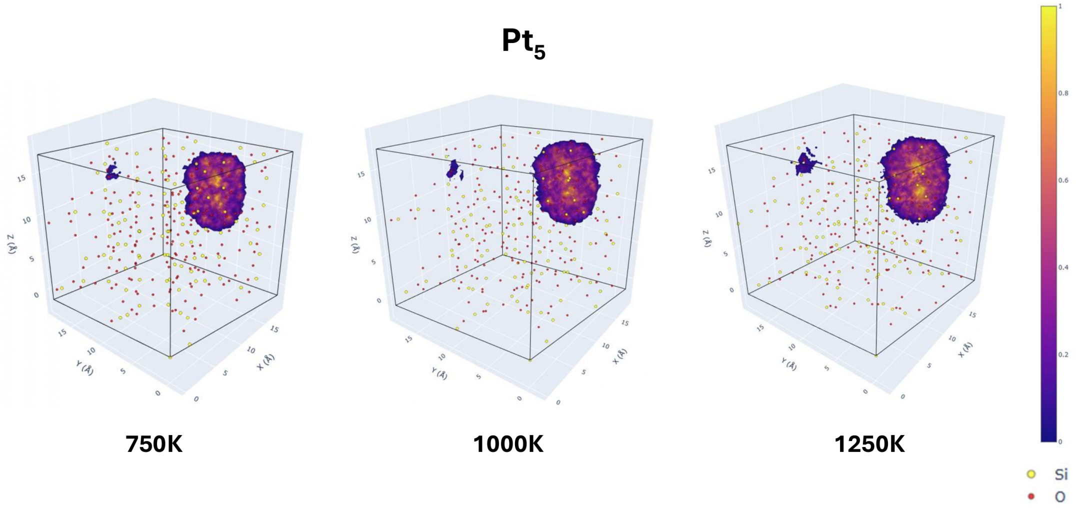

FIGURE 13 Volumetric density maps of Pt_5 (5 Pt atoms in the unit cell) within the CHA zeolite framework at 750 K (left), 1000 K (middle), and 1250 K (right). Si and O atoms of the framework are shown as yellow and red spheres, respectively. The colour bar indicates probability density.

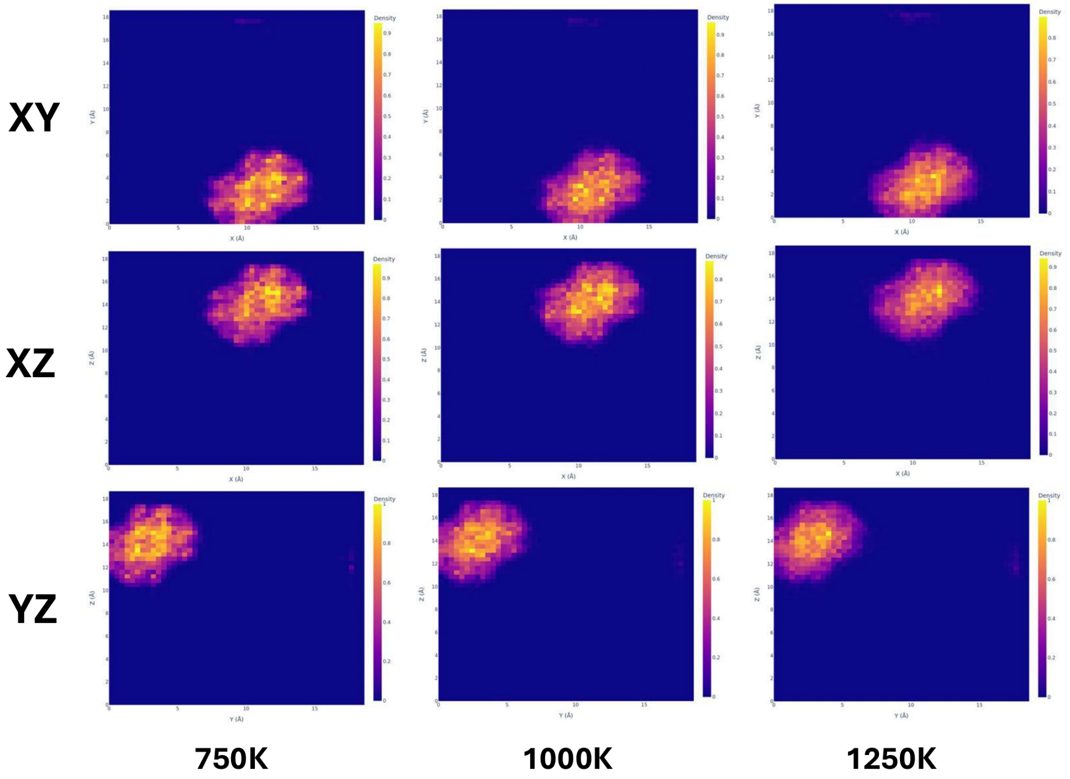

FIGURE 14 2D projections of Pt atomic density within the CHA zeolite framework onto the XY, XZ, and YZ planes at 750 K, 1000 K, and 1250 K. Data obtained from molecular dynamics simulations. The colour bar indicates probability density, highlighting regions of higher Pt concentration.

TABLE 4 Comparative analysis of density features and their relationship to findings in the original study.[23] All energy values and migration barriers are taken from the original work.

Cluster	Volumetric Density Features	Key Findings of Original Work
Pt_1	• Localised in 6MR, no density in cage center<br>• Multi-site hopping at T > 1000K	• Strong bonding to framework<br>• 6MR-hopping barrier: 62 kJ/mol
Pt_3	• Localised in CHA cage (centre)<br>• Density spreads over multiple CHA cages at high-T	• Liquid-like dynamics<br>• 8MR transition barrier: 125 kJ/mol
Pt_5	• Localised in CHA cage centre (even at 1250K)	• No-intercage transitions in 25 ns<br>• 8MR transition barrier: 149 kJ/mol

Comparison and physical insight

The volumetric density analysis quantitatively reproduces key findings from Heard et al.'s neural network potential simulations (also summarised in the Table 4):

- Pt_1: Confinement to 6MR matches the reported one, as the local transitions occur only within the double 6-ring (d6r) unit, but no intercage migration is observed. The migration through the 8MR was reported to be energetically unfavourable (barrier: 111 kJ/mol via NEB calculations) compared to only 62 kJ/mol for local double 6-ring transitions (based on umbrella sampling simulations at 300 K).
- Pt_3: The density is localised predominantly in the CHA cage center, and at 1250K we observe faster and more spread atomic movements resulting in liquid-like dynamics. Also, the intercage transition is more frequent at higher temperature (750K vs 1250K), since cluster has enough (kinetic) energy to overcome the 125 kJ/mol migration barrier. Finally, the largest size of Pt cluster causes increased steric hindrance, limiting their ability of cluster to occupy the double 6-ring units.
- Pt_5: It occupies the center of the CHA cage without any inter-cage transitions observed. This is in line with a large steric hindrance reported for inter-cage transitions (barrier of 149 kJ/mol). Also at increased temperatures Pt_5 clusters show no migration; only an increased motion within cages is observed.

3.4 | Case Study: Bulk Water System

CRISP's post-processing pipeline processed trajectories from Villard et al. [24] and, with just a few high-level commands, computed radial distribution functions, coordination numbers, hydrogen-bond statistics, and mean-square displacements (and associated self-diffusion coefficients) for each trajectory obtained with different meta-GGA DFT functionals.

3.4.1 | Mean–Square Displacement and Diffusion Coefficients

The self-diffusion coefficient (D_L) is determined using the Einstein relation and the equation below in Villard's work[24]:

$$D_L = \frac{1}{6} \lim_{t \to \infty} \frac{d}{dt} \left\langle \frac{1}{N} \sum_{i=1}^{N} |\mathbf{r}_i(t) - \mathbf{r}_i(0)|^2 \right\rangle \tag{16}$$

In this equation, N represents the total number of water molecules, $\mathbf{r}_i(t)$ denotes the position of the oxygen atom for molecule i at time t, and the angle brackets signify an ensemble average within the NVE (constant number of particles, volume, and energy) ensemble. To enhance statistical robustness, MSD values are typically averaged over multiple lag times and time origins. The final D_L value is then derived from the average slope of this mean-squared displacement, obtained using the Diffusion Coefficient Tool plugin for VMD[25, 26].

To assess the overall agreement between the CRISP and VMD method for finite-size diffusion coefficient, we computed the mean absolute error (MAE) across the five functionals:

$$\text{MAE}_{\text{Exp vs. Villard}} = 0.223\ \text{Å}^2/\text{ps}$$
$$\text{MAE}_{\text{Exp vs. CRISP}} = 0.238\ \text{Å}^2/\text{ps}$$

Each MAE is defined as the average of $|D_i - D_j|$ over the five meta-GGA functionals i, comparing (1) experimental vs. simulation values from Villard [24], (2) experimental vs. CRISP-derived values.

The following provides a detailed comparison of the methodological differences between CRISP and the VMD Diffusion Coefficient Tool for extracting diffusion coefficients from molecular dynamics trajectories. These distinctions can influence the resulting values and their statistical reliability, even when analysing identical data. Key aspects are summarised below:

- *Subsampling of lag times:* VMD uses an input-based (restricted by some defaults) range for lag times; CRISP uses all available time origins and lag times by default. Using all lags improves statistics and reduces variance, typically lowering the influence of any rapid motions that can occur before the system reaches steady-state diffusion.
- *Uncertainty estimation:* VMD reports the standard error from a single linear fit to the MSD over the entire interval. In contrast, CRISP calculates the standard error of the mean (SEM) from multiple blockwise D estimates, reflecting variability due to time correlations and the choice of fitting window. This approach provides a more statistically rigorous and realistic measure of uncertainty in the diffusion coefficient.
- *Fitting control:* CRISP allows explicit, user-defined selection of the diffusive interval—including start and end times, optional intercept, block size, and other parameters—enabling precise focus on the truly linear regime of the MSD. While similar adjustments are possible in VMD through multiple attempts in GUI, CRISP offers greater flexibility and reproducibility by leveraging Python scripting, which facilitates more extensive and customizable parameter tuning.
- *Diagnostic Advantages:* CRISP outputs the full MSD time series (CSV), the fitted line over the selected interval, a time-series diffusion coefficient ($D(t)$), and uncertainty estimates for the block fit. These features make it straightforward to report and reproduce the diffusion coefficient. Additionally, CRISP offers enhanced flexibility for handling larger trajectories, supports multiple fitting methods, and enables parallel processing for efficient analysis.

To directly illustrate the impact of analysis choices, we performed a matched comparison using the revM06-L

functional, which showed one of the largest discrepancies between CRISP and VMD in the main Figure 11. For this test, we set CRISP to use the same lag times, fitting interval, and analysis parameters as the VMD Diffusion Coefficient Tool defaults. As shown in Figures 15 and 16, when identical settings are used, CRISP and VMD produce essentially the same diffusion coefficient for the same trajectory. This confirms that the differences observed in the main text are due to specific parameter choices—such as lag times, fit intervals, and analysis ranges between the two analysis approaches.

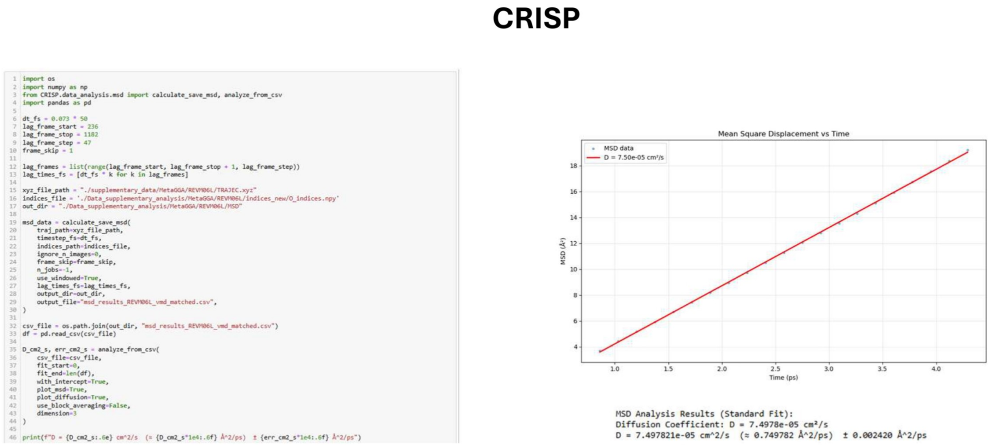

FIGURE 15 CRISP MSD analysis workflow and results for the revM06-L functional. Left: Example Python script showing the configuration and execution of the CRISP MSD analysis module, with lag times from 236 to 1182 frames (step 47), analysis interval from 0 to 2363 frames, and frame timing of 3.65 fs or 0.00000365 ns. Right: Output plot of mean square displacement (MSD) versus time, with the linear fit used to determine the diffusion coefficient. The calculated diffusion coefficient is $D = 0.749782 \pm 0.002420$ Å^2/ps, as shown in the plot.

VMD

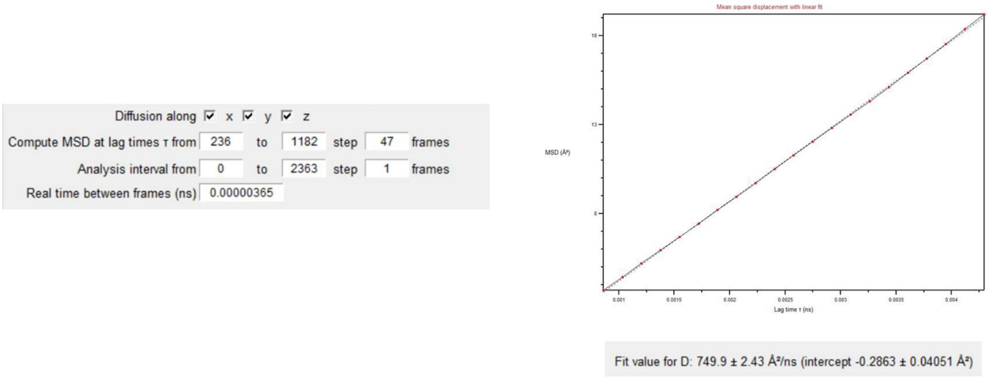

FIGURE 16 VMD Diffusion Coefficient Tool workflow and results for the revM06-L functional. Left: Screenshot of the VMD plugin interface showing user-selected lag times (236 to 1182 frames, step 47), analysis interval (0 to 2363 frames), and frame timing (0.00000365 ns). Right: Output plot of MSD versus lag time with the linear fit, and the resulting diffusion coefficient and intercept values. The calculated diffusion coefficient is $D = 0.7499 \pm 0.00243$ Å^2/ps.

They demonstrate that choices in fitting strategy and other uncertainties regarding the setup are the cause for variations in the calculated diffusion coefficients between these two methods.

3.4.2 | Radial Distribution Function

The comparison of results in a quantitative way (using mean absolute errors (MAEs)) for the various RDF calculations from Villard et al. (using the VMD software[25]) and CRISP; the reference is the experimental RDF data at 298 K, which is shown in grey in Main Figure 12.

TABLE 5 Mean Absolute Errors (MAEs) for RDFs between experimental data and CRISP, and data reported by Villard et al.[24].

RDF Type	MAE (Exp vs. Reported)	MAE (Exp vs. CRISP)
O–O	0.2961	0.2928
O–H	0.5594	0.5594
H–H	0.1954	0.1947

The MAEs for the radial distribution functions (RDFs) calculated using both Villard et al. (via VMD[25]) and CRISP are comparable to the experimental RDF data at 298 K (shown in grey in Main Figure 12). This indicates that both computational approaches yield similar agreement with respect to the experiment. This similarity is further illustrated in the RDF plots, where direct comparisons of O–O, O–H, and H–H RDFs are shown for both methods alongside results gathered from the experiments. These plots demonstrate that both CRISP and VMD approaches produce

consistent RDF profiles for water.

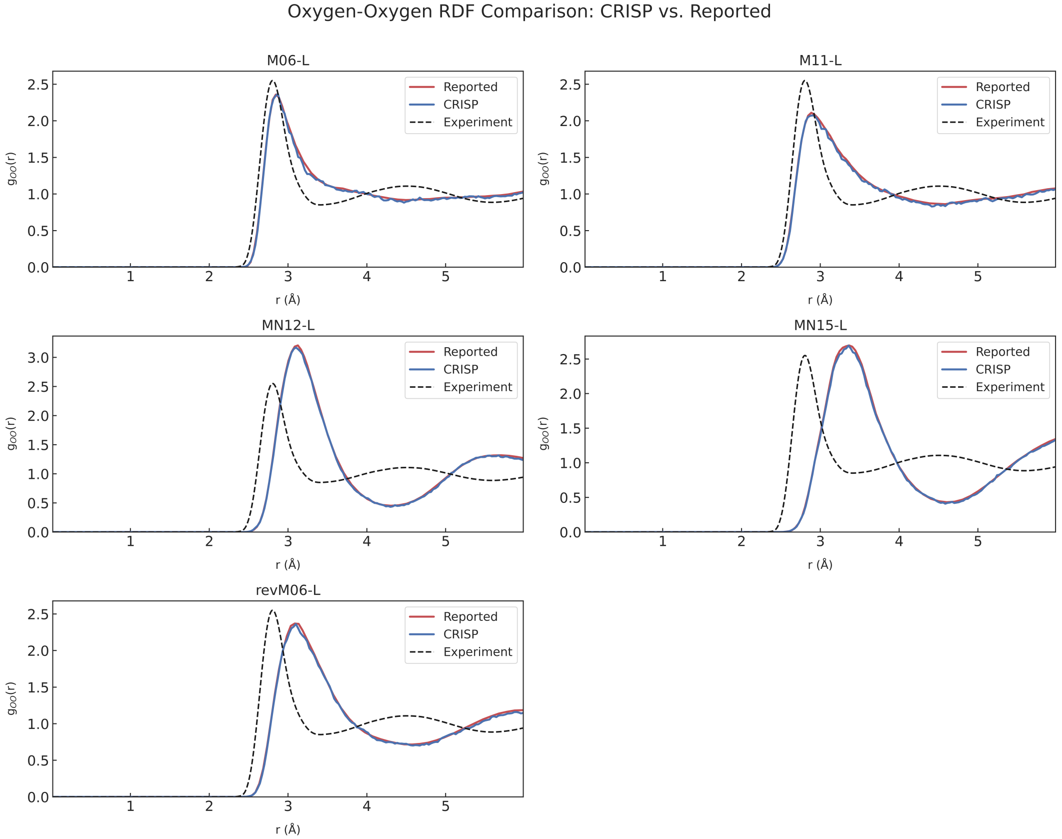

FIGURE 17 A direct comparison of Oxygen-Oxygen (O-O, homo-atom) radial distribution functions (RDFs) for water, calculated using different meta-GGA functionals (M06-L, M11-L, MN12-L, MN15-L, revM06-L) within the CRISP software (in blue line). These RDFs were computed from *ab initio* molecular dynamics trajectories originally sourced from Villard et al.[24]. The results for each functional are compared directly against the computed RDF data from Villard et al., which is represented by the red line.

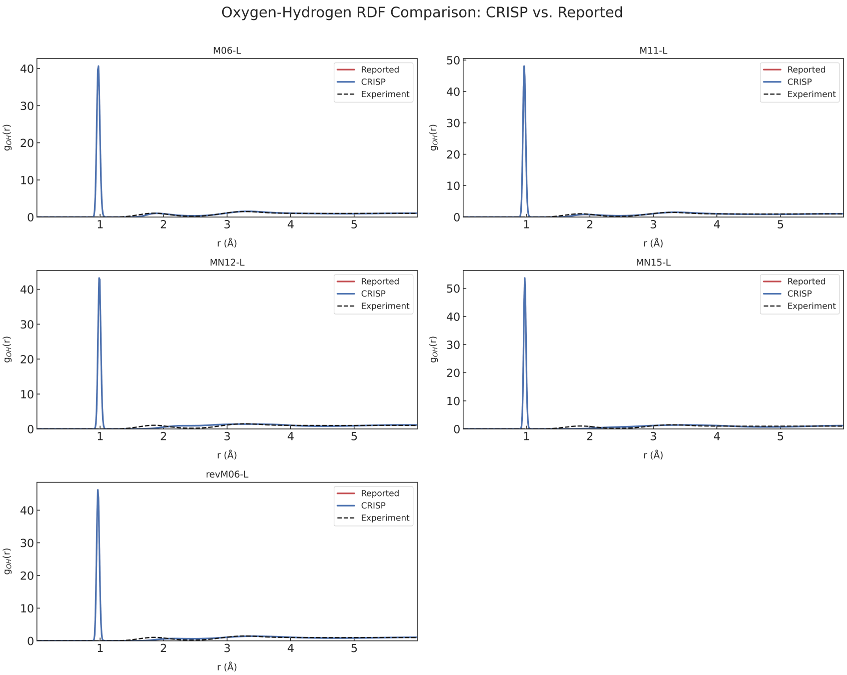

FIGURE 18 A direct comparison of Oxygen-Hydrogen (O-H, hetero-atom) radial distribution functions (RDFs) for water, calculated using different meta-GGA functionals (M06-L, M11-L, MN12-L, MN15-L, revM06-L) within the CRISP software (in blue line). These RDFs were computed from *ab initio* molecular dynamics trajectories originally sourced from Villard et al.[24]. The results for each functional are compared directly against the computed RDF data from Villard et al., which is represented by the red line.

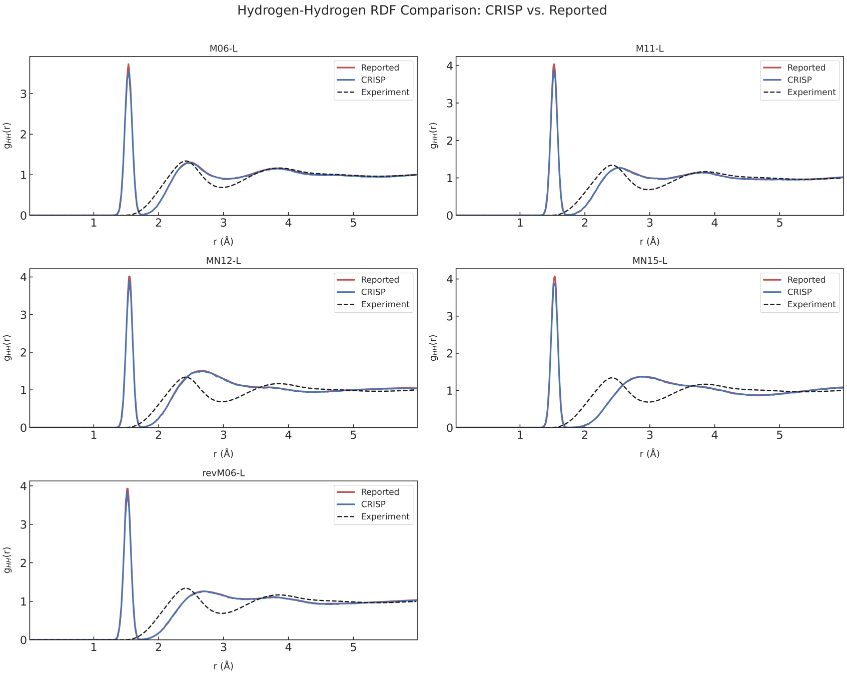

FIGURE 19 A direct comparison of Hydrogen-Hydrogen (H-H, homo-atom) radial distribution functions (RDFs) for water, calculated using different meta-GGA functionals (M06-L, M11-L, MN12-L, MN15-L, revM06-L) within the CRISP software (in blue line). These RDFs were computed from *ab initio* molecular dynamics trajectories originally sourced from Villard et al.[24]. The results for each functional are compared directly against the computed RDF data from Villard et al., which is represented by the red line.

To interpret the structural features of liquid water and assess the reliability of different analysis tools, we compare radial distribution functions (RDFs) calculated using three distinct methods: CRISP, the approach of Villard et al., and ASE. This comparison helps clarify how methodological choices can impact the resulting RDFs, particularly for O–H pairs. As shown in SI Figure 20, the CRISP approach and that of Villard et al. are in close agreement, while the ASE method deviates significantly. This discrepancy arises from the way ASE constructs the atom pairs for RDF calculation: when using `get_rdf(rmax, nbins, elements=['O', 'H'])`, ASE creates a temporary `Atoms` object containing all oxygen and hydrogen atoms, and then computes the RDF between all possible O–H pairs, including extra intramolecular pairs (O–O and H–H) that are not typically considered in standard RDF analysis. This gets omitted when it is calculating for the homo-atoms; therefore, all three methods have better agreement for O-O or H-H pairs. Although there are still some discrepancies that exist that can be attributed to the way ASE normalises. It is using the total number of atoms in the system, rather than the number of target atom pairs (such as O and H atoms). In contrast, the method described in SI Equation 14 uses the number of target atoms for normalisation, as recommended by previous works[27, 28], and is also implemented in VMD[25] and MDAnalysis[29]. The impact of these methodological differences on the O–H RDF for the revM06-L functional is illustrated in Figure below.

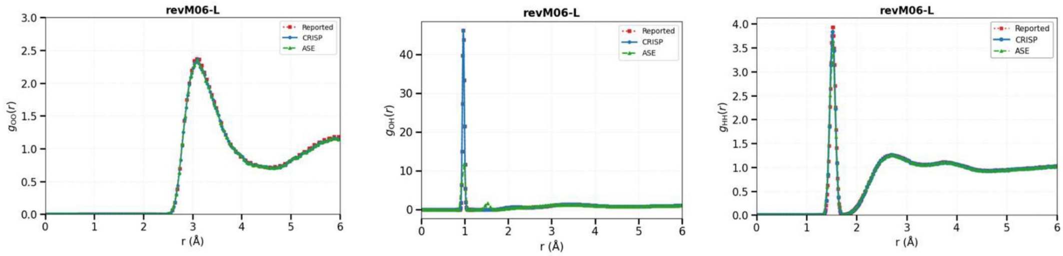

FIGURE 20 Comparison of the Oxygen-Oxygen ($g_{OO}(r)$, left), Oxygen-Hydrogen ($g_{OH}(r)$, middle), and Hydrogen-Hydrogen ($g_{HH}(r)$, right) radial distribution functions (RDFs) of liquid water, calculated with the revM06-L functional. The RDFs obtained from CRISP (blue) and Villard et al.[24] (red) are directly compared against data calculated from ASE (green).

3.4.3 | Coordination Number

To quantify the agreement with experiment and previous computational analysis by Villard et al.[24], we calculated the Mean Absolute Error (MAE) of the oxygen-oxygen coordination number (CN) relative to the experimental value. The "Reported" values refer to those published by Villard et al., where the CN is determined using the first minimum of the O-O radial distribution function (RDF). "CRISP(3.5 Å)" and "CRISP(3.8 Å)" denote CN values calculated using the CRISP tool with fixed cutoff distances of 3.5 Å) and 3.8 Å), respectively. "CRISP(Villard et al.)" refers to CN values calculated using CRISP with a dynamic cutoff approach, matching the method employed by Villard et al. The resulting MAEs are:

$$\mathrm{MAE}_{\text{Exp vs. Reported}} = 4.88$$

$$\mathrm{MAE}_{\text{Exp vs. CRISP}(3.5\ \text{Å})} = 1.82$$

$$\mathrm{MAE}_{\text{Exp vs. CRISP}(3.8\ \text{Å})} = 3.98$$

$$\mathrm{MAE}_{\text{Exp vs. CRISP}(\text{Villardet.al Å})} = 0.10$$

3.4.4 | Hydrogen Bond

The details of the two geometric parameters (oxygen-oxygen distance and donor-hydrogen-acceptor distance) used in the calculations of H-bonds by Villard [24] are as follows:

- **Oxygen-Oxygen Distance ($d = \overline{O_iO_j}$):** The parameters for this function, notably the relevant distance range (2.4 to 3.4 Å), were derived from the first coordination shell of the O-O RDF, directly linking their criterion to experimental structural data.
- **Donor-Hydrogen-Acceptor Distance ($d' = O_iH + HO_j - O_iO_j$):** This metric accounts for the relative orientation and distances of the involved atoms.
  Here, i and j are indices used to distinguish between the two water molecules that are forming a hydrogen bond: one is donating a hydrogen atom for the bond, and the other is accepting it.

Data from Villard's bulk water simulations [24] were used to evaluate the number of average H-bonds per water molecule by CRISP and compared to the values reported by Villard et al. and experimental estimates (see Figure 15 in the main text). Key observations include the following:

- **Overall Similarity:** For most functionals (M06-L, M11-L, MN12-L, revM06-L), the CRISP results (orange bars) closely track the values reported by Villard et al. (green bars) with both being slightly lower than the experimental estimate of 3.8 H-bonds/water (dashed grey line), indicating a reasonably consistent picture of the H-bond network for these functionals, regardless of the precise definition of H-bond used. In both cases, a stark difference appears for MN15-L in which the reported value of the mean H-bond count per water is very low (1.9 from Villard and 2.3 from CRISP) compared to the experiment and other functionals.
  The average difference between experiment and theory is evaluated via Mean Absolute Error (MAE) across all five DFT functionals:

$$\mathrm{MAE}_{\mathrm{Exp\ vs.\ Reported}} = 0.846$$
$$\mathrm{MAE}_{\mathrm{Exp\ vs.\ CRISP}} = 0.680$$

3.5 | Validation and Benchmarking Against Established Tools

To ensure numerical consistency and methodological reliability, we present comprehensive quantitative validation of CRISP analyses against established computational methods (ASE native modules, VMD workflows, and other implementations documented in Villard's peer-reviewed literature[24]).

3.5.1 | Radial Distribution Functions and Performance Metrics

Radial Distribution Function (RDF) calculations were compared against the VMD-based methodology reported by Villard et al. (2024) for identical bulk water (D_2O) trajectories generated using five meta GGA exchange correlation functionals: M06-L, M11-L, MN12-L, MN15-L and revM06-L. Table 6 reports the Mean Absolute Errors (MAE) between experimental X-ray and neutron diffraction data at 298 K and computed RDFs for three atom pair types.

CRISP results match VMD outputs to machine precision across all three pair types. Direct overlay of CRISP RDFs against both VMD and experimental reference data confirms numerical equivalence and validates the `prdf.py` module

TABLE 6 RDF validation: CRISP vs. VMD method against experimental data (Villard et al., 2024).

Pair Type	MAE (VMD)	MAE (CRISP)	Difference
O–O	0.2961	0.2928	0.0033
O–H	0.5594	0.5594	0.0000
H–H	0.1954	0.1947	0.0007

implementation. Notably, comparison with the native RDF method in ASE (SI Figure 20) reveals systematic deviations in O-H pairs. This discrepancy arises from ASE's incorrect handling of intramolecular pair normalisation. Specifically, the `get_rdf()` function in ASE includes unwanted intramolecular O-H pairs and normalises by total atom count rather than target atom species count. This violates standard normalisation conventions adopted by VMD, MDAnalysis and CRISP.

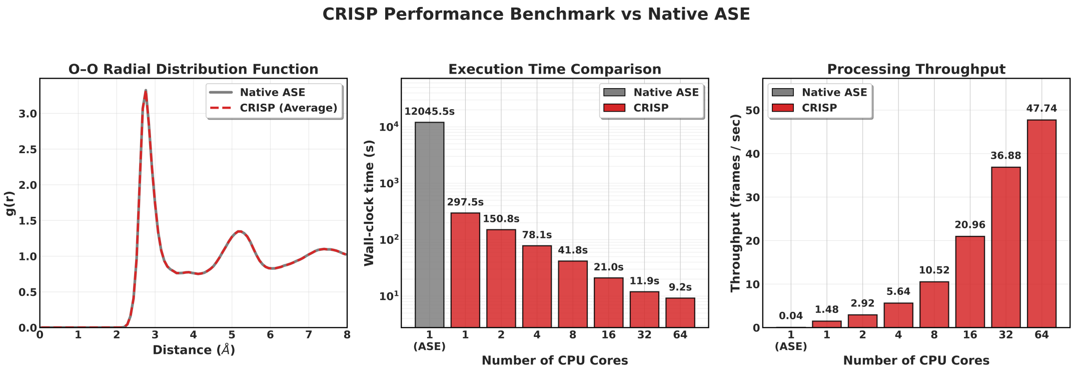

FIGURE 21 Performance and numerical consistency benchmark of native ASE versus the CRISP (`prdf` module) implementation. The analysis calculates the Oxygen-Oxygen Radial Distribution Function (RDF) for a 1,248-atom FAU aluminosilicate system over a ≈ 2 ns trajectory. Scaling tests were performed on an AMD EPYC 9554 workstation, ranging from 1 to 64 CPU cores.

Figure 21 presents the direct results of a benchmark conducted to evaluate the numerical accuracy and computational performance of CRISP against native ASE. The Oxygen-Oxygen atomic pairs were selected for this RDF test to provide a reliable baseline because the native ASE implementation contains a known normalisation bug for heteroatom pairs (discussed previously in Figure 20). The benchmarked system consists of 1,248 atoms within a water-loaded FAU aluminosilicate framework, tracked over a trajectory of 44,000 total frames representing ≈ 2 ns of simulation. From this trajectory, 440 frames were analysed using a skip of 100.

As illustrated in the left panel of Figure 21, the RDF profiles produced by CRISP and native ASE are numerically identical, which confirms that the parallel implementation of the code in CRISP strictly preserves the physical accuracy of the underlying simulation data.

Crucially, the performance metrics reveal that CRISP delivered a significant reduction in execution time. By transitioning from the sequential processing of native ASE to CRISP, the wall clock time decreased from 12045.5 seconds to 9.2 seconds (on 64 cores). This represents a cumulative speedup of over 1300 times, elevating the processing throughput from 0.04 to 47.74 frames per second and demonstrating the efficiency of CRISP for large-scale trajec-

tory analysis.

3.5.2 | Mean Squared Displacement and Diffusion Coefficients

Mean Square Displacement (MSD) calculations and resulting diffusion coefficients were rigorously validated across all five meta GGA functionals against the VMD Diffusion Coefficient Tool workflow. All calculations utilise the Einstein relation:

$$D_L = \frac{1}{6} \lim_{t \to \infty} \frac{d}{dt} \left\langle \frac{1}{N} \sum_{i=1}^{N} |\mathbf{r}_i(t) - \mathbf{r}_i(0)|^2 \right\rangle \tag{17}$$

Table 7 reports diffusion coefficients (D) for the revM06-L functional. When identical settings for lag times and fitting intervals are applied, results match to high precision.

TABLE 7 MSD validation: Finite-size diffusion coefficient agreement (revM06-L example)

Method	D (Å^2/ps)	Uncertainty
CRISP	0.7498	±0.0024
VMD Tool	0.7499	±0.0024

Methodological differences between the tools can involve fitting control and uncertainty estimation. While VMD utilises a single linear fit, CRISP offers a more statistically rigorous approach by calculating the Standard Error of the Mean (SEM) from multiple blockwise D estimates. When CRISP is configured with identical lag times and fitting windows as VMD (SI Figures 15, 16), results match to machine precision. This validates the `msd.py` module implementation.

3.5.3 | Coordination Number Analysis

Validation of the `contact_coordination.py` module was performed by comparing Oxygen-Oxygen coordination numbers (CN) against experimental benchmarks (4.3 ± 0.2) and results reported by Villard et al. We evaluated with dynamic cutoffs based on the first minimum of the radial distribution function (RDF) as stated to be done by Villard with VMD. The small discrepancy between the values can be attributed to judging the minimum values (M06-L: 2.85Å, M11-L: 2.91Å, MN12-L: 3.09Å, MN15-L: 3.33Å, revM06-L: 3.09Å), since the judged minimum values of the original work were not explicitly mentioned.

To assess the precision of our implementation, we compared the CN values reported using VMD against those calculated via the CRISP method using the Villard cutoff criteria. The functional specific results for the coordination number comparison are presented in Table 8.

The CRISP method reproduces the trends observed in the VMD-reported values with a high degree of fidelity. The very good agreement ($\text{MAE} = 0.10$) between CRISP and the reported values when using the dynamic Villard cutoff confirms the algorithmic consistency of our implementation with established literature. This small discrepancy is attributed to minor differences in the dynamic determination of the first RDF minimum between the two software packages.

TABLE 8 Comparison of Oxygen-Oxygen coordination numbers: Reported (VMD) vs. CRISP (Villard Cutoff).

Functional	CN (Reported)	CN (CRISP)	Difference
M06-L	4.9	4.86	0.04
M11-L	6.9	6.80	0.10
MN12-L	11.2	11.09	0.11
MN15-L	13.0	12.88	0.12
revM06-L	9.9	9.76	0.14

3.5.4 | Hydrogen Bond Statistics

Finally, we validated the `h_bond` module by comparing the average number of hydrogen bonds per water molecule. Villard et al. utilised a smoothed probability function based on two geometric parameters: Oxygen-Oxygen distance and Donor-Hydrogen-Acceptor distance. In contrast, CRISP employs established IUPAC geometric criteria based on distance and angle cutoffs. Despite these different parameterisations, both methods capture identical trends across the meta GGA functionals. For most functionals, including M06-L, M11-L and MN12-L, CRISP results closely track reported values.

TABLE 9 Hydrogen bond validation: average number of H-bonds per water molecule across meta-GGA functionals. Experimental estimate: 3.8 H-bonds/water at 298 K

Functional	Reported (VMD)	CRISP	Difference
M06-L	3.48	3.504	0.024
M11-L	3.22	3.377	0.157
MN12-L	3.24	3.388	0.148
MN15-L	1.93	2.270	0.340
revM06-L	2.90	3.059	0.159
**Statistical Summary:**			
$\text{MAE}_{\text{Exp vs. Reported}}$ = 0.846 H-bonds/water			
$\text{MAE}_{\text{Exp vs. CRISP}}$ = 0.680 H-bonds/water			

The lower MAE for CRISP suggests that the direct geometric definition based on IUPAC criteria provides a reliable and potentially more accurate measure of hydrogen bond networks compared to smoothed probability functions derived from RDF minima. This advantage likely stems from CRISP's explicit consideration of both distance and angular criteria, which more closely reflect the directional nature of hydrogen bonding interactions.

3.5.5 | Numerical Stability and Edge Case Testing

The CRISP test suite has 81% code coverage, uses a comprehensive pytest framework with many modules and functions tested. It validates:

- **Small systems**: Verified RDF and MSD calculations on systems with $N < 50$ atoms (numerical stability at low statistics)
- **Boundary conditions**: Tested triclinic and orthorhombic cells with non-90° angles (minimum image convention robustness)
- **Frame subsampling**: Validated frame skipping with stride values up to 100 frames (memory mapping consistency)
- **Empty selections**: Tested atom index selection yielding zero atoms (error handling and graceful degradation)

All validation code and benchmark scripts are provided in the repository (`examples/validation/`) with detailed methodology documentation and reproducibility instructions.

references

[1] Odoh SO, Deem MW, Gagliardi L. Preferential Location of Germanium in the UTL and IPC-2a Zeolites. The Journal of Physical Chemistry C 2014;118(46):26939–26946.

[2] Rojas A, Camblor MA. A pure silica chiral polymorph with helical pores. Angewandte Chemie (International Ed in English) 2012;51(16):3854–3856.

[3] Allen MP, Tildesley DJ. Computer simulation of liquids. Oxford university press; 2017.

[4] Bartók AP, Kondor R, Csányi G. On representing chemical environments. Physical Review B 2013 5;87(18). http://dx.doi.org/10.1103/PhysRevB.87.184115.

[5] Seabold S, Perktold J. statsmodels: Econometric and statistical modeling with python. In: 9th Python in Science Conference; 2010. .

[6] Inc PT, Collaborative data science. Montreal, QC: Plotly Technologies Inc.; 2015. https://plot.ly.

[7] Ester M, Kriegel HP, Sander J, Xu X, et al. A density-based algorithm for discovering clusters in large spatial databases with noise. In: kdd, vol. 96; 1996. p. 226–231.

[8] Rousseeuw PJ. Silhouettes: a graphical aid to the interpretation and validation of cluster analysis. Journal of computational and applied mathematics 1987;20:53–65.

[9] Arunan E, Desiraju GR, Klein RA, Sadlej J, Scheiner S, Alkorta I, et al. Definition of the hydrogen bond (IUPAC Recommendations 2011). Pure and Applied Chemistry 2011 jul 8;83(8):1637–1641. http://dx.doi.org/10.1351/PAC-REC-10-01-02.

[10] Einstein A. Über die von der molekularkinetischen Theorie der Wärme geforderte Bewegung von in ruhenden Flüssigkeiten suspendierten Teilchen. Annalen der Physik 1905 1;322(8):549–560. http://dx.doi.org/10.1002/andp.19053220806.

[11] Hansen JP, McDonald IR. Theory of Simple Liquids. Elsevier Academic Press; 2006.

[12] Giorgino T. Computing 1-D atomic densities in macromolecular simulations: The density profile tool for VMD. Computer Physics Communications 2014;185(1):317–322.

[13] Eldar Y, Lindenbaum M, Porat M, Zeevi YY. The farthest point strategy for progressive image sampling. 12th IAPR International Conference on Pattern Recognition, 1994, IEEE;. p. 93–97. http://dx.doi.org/10.1109/ICPR.1994.577129.

[14] Laakso J, Himanen L, Homm H, Morooka EV, Jäger MO, Todorović M, et al. Updates to the DScribe library: New descriptors and derivatives. The Journal of Chemical Physics 2023;158(23).

[15] Wilson GT. Time Series Analysis: Forecasting and Control, 5th Edition, by George E. P. Box, Gwilym M. Jenkins, Gregory C. Reinsel and Greta M. Ljung, 2015. Published by John Wiley and Sons Inc., Hoboken, New Jersey, pp. 712. ISBN: 9781118675021. Journal of Time Series Analysis 2016 mar 27;37(5):709–711. http://dx.doi.org/10.1111/jtsa.12194.

[16] Sokal A. In: DeWitt-Morette C, Cartier P, Folacci A, editors. Monte Carlo Methods in Statistical Mechanics: Foundations and New Algorithms Boston, MA: Springer US; 1997. https://doi.org/10.1007/978-1-4899-0319-8_6.

[17] Bondi Av. van der Waals Volumes and Radii. The Journal of physical chemistry 1964;68(3):441–451.

[18] Ester M, Kriegel HP, Sander J, Xu X. A density-based algorithm for discovering clusters in large spatial databases with noise. In: Proceedings of the Second International Conference on Knowledge Discovery and Data Mining KDD'96, AAAI Press; 1996. p. 226–231.

[19] Usler AL, Kemp D, Bonkowski A, De Souza RA. A general expression for the statistical error in a diffusion coefficient obtained from a solid-state molecular-dynamics simulation. Journal of Computational Chemistry 2023;44(14):1347–1359.

[20] Willimetz D, Erlebach A, Heard CJ, Grajciar L. ^{27}Al NMR chemical shifts in zeolite MFI *via* machine learning acceleration of structure sampling and shift prediction. Digital Discovery 2025;4(1):275–288. http://dx.doi.org/10.1039/D4DD00306C.

[21] Batatia I, Benner P, Chiang Y, Elena AM, Kovács DP, Riebesell J, et al., A foundation model for atomistic materials chemistry; 2024. https://arxiv.org/abs/2401.00096.

[22] Christensen AS, von Lilienfeld OA, On the role of gradients for machine learning of molecular energies and forces; 2020. https://arxiv.org/abs/2007.09593.

[23] Heard CJ, Grajciar L, Erlebach A. Migration of zeolite-encapsulated subnanometre platinum clusters via reactive neural network potentials. Nanoscale 2024;16(16):8108–8118.

[24] Villard J, Bircher MP, Rothlisberger U. Structure and dynamics of liquid water from ab initio simulations: adding Minnesota density functionals to Jacob's ladder. Chemical Science 2024;15(12):4434–4451.

[25] Humphrey W, Dalke A, Schulten K. VMD: visual molecular dynamics. Journal of molecular graphics 1996;14(1):33–38.

[26] Giorgino T. Computing diffusion coefficients in macromolecular simulations: the Diffusion Coefficient Tool for VMD. Journal of Open Source Software 2019;4(41):1698.

[27] Hansen JP, McDonald IR. Theory of simple liquids: with applications to soft matter. Academic press; 2013.

[28] Levine BG, Stone JE, Kohlmeyer A. Fast analysis of molecular dynamics trajectories with graphics processing units—Radial distribution function histogramming. Journal of computational physics 2011;230(9):3556–3569.

[29] Michaud-Agrawal N, Denning EJ, Woolf TB, Beckstein O. MDAnalysis: a toolkit for the analysis of molecular dynamics simulations. Journal of computational chemistry 2011;32(10):2319–2327.
